# Spatio-temporal variations in bacterial and fungal community associated with dust aerosol in Kuwait

**DOI:** 10.1371/journal.pone.0241283

**Published:** 2020-11-05

**Authors:** Fadila Al Salameen, Nazima Habibi, Saif Uddin, Khalil Al Mataqi, Vinod Kumar, Bashayer Al Doaij, Sami Al Amad, Ebtisam Al Ali, Faiz Shirshikhar

**Affiliations:** 1 Biotechnology Program, Environment and Life Sciences Research Centre, Kuwait Institute for Scientific Research, Shuwaikh, Kuwait; 2 Environment and Climate Change Program, Environment and Life Sciences Research Centre, Kuwait Institute for Scientific Research, Shuwaikh, Kuwait; University of Maine, UNITED STATES

## Abstract

Kuwait is a country with a very high dust loading; in fact it bears the world’s highest particulate matter concentration in the outdoor air. The airborne dust often has associated biological materials, including pathogenic microbes that pose a serious risk to the urban ecosystem and public health. This study has established the baseline taxonomic characterization of microbes associated with dust transported into Kuwait from different trajectories. A high volume air sampler with six-stage cascade impactor was deployed for sample collection at a remote as well as an urban site. Samples from three different seasons (autumn, spring and summer) were subjected to targeted amplicon sequencing. A set of ~ 50 and 60 bacterial and fungal genera, respectively, established the core air microbiome. The predominant bacterial genera (relative abundance ≥ 1%) were *Brevundimonas* (12.5%), *Sphingobium* (3.3%), *Sphingopyxis* (2.7%), *Pseudomonas* (2.5%), *Sphingomonas* (2.4%), *Massilia* (2.3%), *Acidovorax* (2.0%), *Allorhizobium* (1.8%), *Halomonas* (1.3%), and *Mesorhizobium* (1.1%), and the fungal taxa were *Cryptococcus* (12%) followed by *Alternaria* (9%), *Aspergillus* (7%), *Candida* (3%), *Cladosporium* (2.9%), *Schizophyllum* (1.6%), *Fusarium* (1.4%), *Gleotinia* (1.3%) and *Penicillium* (1.15%). Significant spatio-temporal variations were recorded in terms of relative abundances, α-diversities, and β-diversities of bacterial communities. The dissimilarities were less pronounced and instead the communities were fairly homogenous. Linear discrimant analysis revealed three fungal genera known to be significantly differentially abundant with respect to different size fractions of dust. Our results shed light on the spatio-temporal distribution of airborne microbes and their implications in general health.

## Introduction

Dust storm episodes are among the most important weather phenomena in arid countries around the world [1, 2]. They are caused by high-energy winds eroding the topsoil in regions with minimal vegetation cover. Kuwait and other countries in the Middle East experience some of the worst dust storm episodes around the world. Kuwait is particularly susceptible to dust storms because of its low topography, scant vegetative cover, and strong turbulent winds that occur particularly in the summer months [3–5]. The rates of dust fallout in Kuwait have been reported to be amongst the highest in the world with mean monthly concentrations as high as1400 μg m^-3^ [6].

Due to the high concentration of contaminants associated with dust; the increasing frequency of dust storms pose a potential health hazard for humans [7, 8]. Those particles also include biological materials like fungal spores, bacteria, viruses and fragments from plants (pollen) and animals [9]. The abundance of microorganisms in the aerosols highlight the health hazards as a result of inhalation, since the dust serves as carriers for pathogens and allergens acting as a vector for pulmonary/respiratory infections [10].

A few studies completed in Asia, North America, and the Middle East have addressed the issue of the impact of dust storms on human health [11–14]. The bacteria and fungi associated with dust have been associated with the development of hypersensitive responses, asthma, allergic reactions, infections, and toxicosis of the respiratory system in humans [12, 15, 16]. Moreover, the microbial molecules such as endotoxins and mycotoxins can cause respiratory stress, fever, and flu-like illnesses which prove to be fatal [17]. It was recorded that the outbreaks of Valley Fever within the United States (in the early 1990s) were associated with dust storms [18]. The World Health Organization (WHO) found that dust storm activity was the main cause of outbreaks of bacterial meningitis in sub-Saharan Africa [19]. The plant and human pathogen bacteria were identified and characterized from dust events in the Virgin Islands and Mali (West Africa) [20, 21]. Griffin [22], reported pneumonia from dust storm exposure in the Middle East, especially those cases of deployed military personnel.

A study conducted on aeroallergens and their relationship with asthma-related visits to hospitals in Kuwait [23, 24] established increased admission due to elevated pollen counts in the air. Two studies demonstrated that several countries in the Gulf region were impacted by desert dust storms such as Kuwait and Saudi Arabia. This was manifested through the reporting of a higher prevalence of asthma, particularly in the pediatric population, when compared with European countries [25]. Thalib and Al-Taiar [14] also reported that dust storm events in Kuwait have a significant impact on respiratory and asthma admission.

Although the study of dust materials has attracted many researchers around the world, information about the biological content of dust in Kuwait and the Arabian Gulf region is still limited. There is no information available in the current literature on microbes that are associated with different size fractions of the dust and the taxonomic variations, if any, with the respirable and inhalable fractions of airborne dust. This study presents a baseline for the microbial composition of the airborne dust and its spatio-temporal variations in Kuwait. Bacterial and fungal populations associated with different aerosol size fractions have been identified through the advanced methods of next-generation sequencing.

## Materials and methods

### Sampling

Two sampling sites were chosen on the basis of dominant wind direction. The first one was a remote location in Abdally (30.05 N 47.71 E; 21m above sea level) where the air mass enters into Kuwait (S1 Fig), and the second was a urban site in Kuwait City (29.34 N 47.91 E; 1 m above sea level) where the air mass follows the northwest-southeast wind direction (Table 1). The field data collection at both sites was carried out in accordance with the procedure laid down by the Kuwait Institute for Scientific Research for collection of field samples. The remote site was a private farm belonging to Dr. Hasan Al-Shammari, who has allowed the installation of the High Volume Air Sampler (HVAS) and supplied the electricity for its operation throughout the duration of the study. The urban site is located within the premises of the Kuwait Institute for Scientific Research, and the sampler was installed with due permission of the authorities. At both sites, we have been provided free access and ownership of the samples and data obtained. These sites have no protected species, one being an agriculture farm and the other is office premises. The aerosol samples were collected using a HVAS equipped with a six-stage cascade impactor. The air was drawn at the rate of 566 L min^-1^ with the aid of a pump that had a clean, sterilized, Tisch-slotted quartz filter (5.25” x 5.5”) in each size fraction, and a Whatman GFF (8” x 10”) at the end to trap particulate matter as fine as 0.39 μm (between the aerodynamic diameter (Dp) sizes ranges of < 0.39 (backup high volume filter-Stage 7), 0.39 to 0.69 (Stage 6), 0.69 to 1.3 (Stage 5), 1.3 to 2.1 (Stage 4), 2.1 to 4.2 (Stage 3), 4.2 to 10.2 (Stage 2) and > 10.2 μm (Stage 1) for a period of 48 hrs (1632 ± 10 m^3^). The exact air volume for each sample was determined with the use of a calibrated Magnehelic gauge (Tisch Environmental, Inc.) for measuring the pressure at the start and end of each sampling period. A particle mass counter was co-deployed with HVAS at each site to have an additional verification of the particle mass and count. The samples were collected for three seasons at both sites to capture the spatio-temporal variability. Instruments were calibrated as per the manufacturer’s guidelines.

**Table 1:** Sample details of dust specimens collected during five time points during three seasons from two sites in Kuwait. Sampling performed through high stage cascade impactor.

Sample collection	Site	Elevation	GPS coordinates	Season	*Impactor Stage	
					Bacterial detection	Fungal detection
October, 2017	Remote	21	30.05 N 47.71 E	Autumn	2,3,5	3,4
	Urban	1	29.34 N 47.91 E		4,5	4,5
April, 2018	Remote	21	30.05 N 47.71 E	Spring	1,3,4	1,2,3
	Urban	1	29.34 N 47.91 E		1,2,3,4	1,2,3,4,7
June, 2018	Remote	1	29.34 N 47.91 E	Summer	1,2,3,4,5	1,2,5
July, 2018	Urban	21	30.05 N 47.71 E	Summer	3,4,5	3,4,5,7
August, 2018	Remote	21	30.05 N 47.71 E	Summer	-	1

*Numbers 1–7 represent the stage of the impactor. Dust fractions impacted on stage 1–5 fall under the respirable category, whereas stage 7 represents the inhalable portion of the air-borne dust. Sampling was done for a period of 48 hours. The total suspended PM in each size fraction were between the aerodynamic diameter (Dp) sizes ranges of < 0.39 (backup high volume filter-stage 7), 0.39 to 0.69 (stage 6), 0.69 to 1.3 (stage 5), 1.3 to 2.1 (stage 4), 2.1 to 4.2 (stage 3), 4.2 to 10.2 (stage 2) and > 10.2 μm (stage 1).

### DNA isolation and PCR amplification

The total DNA was successfully extracted from dust-laden filter papers using the Wizard® Genomic DNA Purification kit (Promega, Madison, WI). A clean filter paper (without any dust on it) was simultaneously processed for DNA isolation as a negative control. Filter papers with the embedded dust were cut and immersed into 30 ml of sterile phosphate-buffered saline (PBS) for 24 hrs to collect the dust sediment following high-speed centrifugation (20,000 rpm) for 30 mins. The collected dust pellet was lysed and DNA was purified as per the manufacturer’s instructions. The DNA quantification process was performed by a Qubit fluorometer (Invitrogen, CA). Standard Illumina primers were used to amplify the V3 and V4 regions of the 16S rRNA gene and the ITS1and ITS2 regions for bacterial and fungal population identification, respectively [26]. The PCR reaction mixtures (25 μl) contained 2.5 μl of sample DNA, 12.5 μl of 2x KAPA HiFi HotStart ReadyMix (Kapa Biosystems, Boston, MA), and 5 μl each of forward and reverse primer. Standard internal positive (Bacterial-*E*.*coli*-1ng/μl; Fungal-*S*.*cerevisae* 1ng/μl) and negative controls (Nuclease free water) were used in all PCR reactions. The reaction was carried out in Veriti Thermal Cyclers (Applied Biosystems, Grand Island, NY) with initial activation of the DNA polymerase at 95°C for 3 min, followed by 35 cycles each for 30 s at 95°C, 55°C, and 72°C with a final extension step at 72°C for 5 min. The PCR products were visualized on 1.8% agarose gel that was run at 8V/cm for 1 h. Gel images were documented using the gel documentation system (Chemidoc MP, BioRad, USA).

### Targeted amplicon (16s rRNA/ITS) sequencing and analysis

A total of 5ng of PCR product was used for library preparation using the NEBNext Ultra DNA library preparation kit (New England BioLabs, France). The library quantification and quality estimation were done in Agilent 2200 TapeStation (Santa Clara, CA). The prepared library was sequenced in Illumina HiSeq 2500 (San Diego, CA) with 2*250 cycle chemistry. The libraries were spiked with a minimal concentration of 5% PhiX as a positive control. The raw reads were demultiplexed and quality checked employing FastQC (version.0.11.8). In-house PERL scripts were used to trim the primers. The reads were merged using FLASH program (version 1.2.11) with a minimum overlap of 10bp to the maximum overlap of 240bp with zero mismatches yielding an average contig length of 350 to 450bp. The raw reads were subjected to chimera removal using the de-novo chimera removal method UCHIME (version 11) implemented in the tool VSEARCH. Pre-processed reads from all samples were pooled and clustered into operational taxonomic units (OTUs) based on their sequence similarity using the Uclust program (similarity cutoff = 0.97) available in QIIME (Version: 1.9.1) queried against the Greengenes 13_8 taxonomy classifier [26]. OTUs with less than 5 reads were removed (S1a Table in S1 Appendix), and remaining OTUs (~ 2200) were selected for subsequent analysis [26].

### Data visualization and statistical analysis

QIIME generated *.biome files (S1 and S2 Files) were exported to the MicrobiomeAnalyst for further statistical analysis and data visualization through the Marker Data Profiling (MDP) module [27]. The online module additionally filtered OTUs with ≥2 counts leaving a total of 1874 and 1330 OTUs bacterial and fungal samples respectively. Low count (Sample Prevalence-20%) and Low variance filter (Inter-Quartile range-10%) removed additional (bacterial-1624+26; fungal 921+42) OTUs retaining 224 (bacterial) and 371 (fungal) high-quality OTUs for subsequent interpretations employing the necessary statistical parameters (S1b Table in S1 Appendix).

Data rarefaction was performed and the rarefaction curves plotted with a Good’s coverage *ca*.98% for all the samples confirming an adequate sampling of true biodiversity of individual specimens (S1a, S1b in S1 Appendix). Thereafter taxonomic classification was depicted through the differential tree analysis employing the Wilcoxon rank test (P < 0.05) on median abundances. A core microbiome analysis was also performed with a sample prevalence set at 20% and RA of 0.01. The hierarchical clustering was performed on Euclidean distances using the Ward algorithm. Six alpha diversity parameters (Observed, Chao1, ACE, Shannon, Simpson, and Fisher) were compared at all the experimental features through Student’s t-test or ANOVA (Analysis of Variance) at a p value cut off set at P < 0.05. To identify the significant features at three experimental factors (site, season and impactor stage), a total sum normalization (TSN) was performed on the filtered OTUs and the LEfSe algorithm was employed for the linear discriminant analysis (LDA) at P ≤ 0.05 and an adjusted false discovery rate (FDR/q-value) set at 0.05. The permutational multivariate analysis of variance (PERMANOVA) and analysis of similarities (ANOSIM) statistical approaches on BRAY Curtis distances were applied for the Principal Coordinate Analysis (PCoA) on data normalized through the centered log-ratio (CLR) [27].

## Results

### Taxonomic distribution and core microbiome

**Fig 1:**
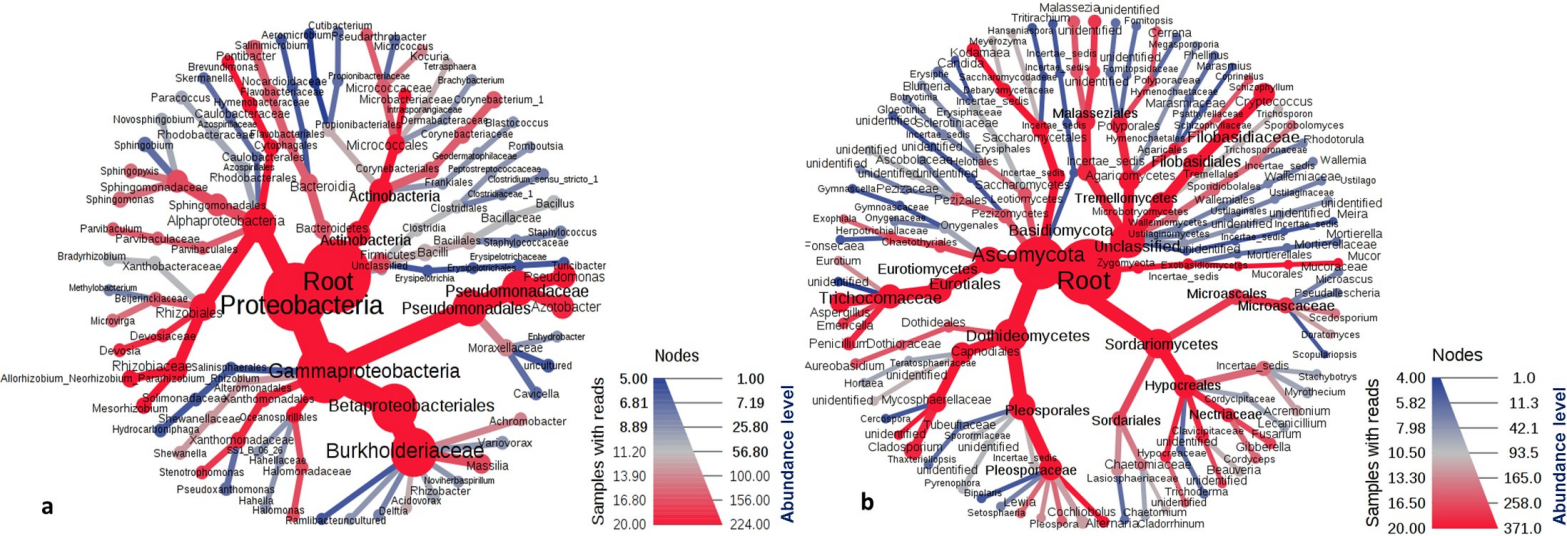
Taxonomic distribution of microbial populations associated with dust aerosols in Kuwait, (a) Bacterial; (b) Fungal. A differential heat tree was plotted to depict the commonly abundant taxa in terms of relative abundance. The nodes in red highlight the constantly detected genera, families, orders, classes and phyla.

Entire taxonomic classification of the bacterial and fungal populations representing the most abundant taxa in terms of their relative abundances (Phyla, Class, Order, Family, Genus) is shown on a heat tree (Fig 1A and 1B). At the phylum level an overwhelming relative abundance (RA) of *Proteobacteria* (98%) was observed. After the phyla, the maximum dominance at the class level was documented for *Gammaproteobacteria* (71%) followed by *Alphaproteobacteria* (27%). The classes were divided into 22 orders, with the top ones being *Betaproteobacteriales* (43%), *Pseudomonadales* (24%), *Caulobacterales* (13%), *Sphingomonadales* (9.3%), *Rhizobiales* (4%), *Oceanospirillales* (1.4%) and *Xanthomonadales* (1.1%). Subsequently, 34 families were found, and the list was topped by *Burkholderiaceae* (43%), *Pseudomonadaceae* (22%), *Caulobacteraceae* (13%), *Sphingomonadaceae* (9%).

Further classification up to the genus level revealed the occurrence of 50 taxons (RA- 0.1 to 58.5%) with the unassigned bacteria dominating the list. Among the classified genera the uppermost RA of 12.5% was recorded for *Brevundimonas* followed by *Sphingobium* (3.3%), *Sphingopyxis* (2.7%), *Pseudomonas* (2.5%), *Sphingomonas* (2.4%), *Massilia* (2.3%), *Acidovorax* (2.0%), *Allorhizobium* (1.8%), *Halomonas* (1.3%) and *Mesorhizobium* (1.1%). The RA of each taxonomic level is presented in S1 Table. As for the core microbiome analysis, the prevalence of these taxa ranged from 0–1 in all the samples.

In the case of the fungal biome, the maximum RA of phyla were recorded for *Ascomycota* (64%). The RA of class was led by *Dothideomycetes* (35%). A total of 28 orders were identified in the present study with the most prevalent one being *Capnodiales* (19%). Moving forward towards the family classification, the most common was of *Mycosphaerellaceae* (18%) followed by *Filobasidiaceae* and *Pleosporaceae* at 12% and 11%, respectively. In addition to the above classification, out of 337 OTUs 68 were classified as a genus. The OTUs that existed in QIIME databases but did not have a taxonomic rank were all together categorized as unassigned (32%), whereas those nonexistent in the software were named as ‘unidentified’ (19%). Among the classified taxa the highest RA were recorded for *Cryptococcus* (12%) followed by *Alternaria* (9%), *Aspergillus* (7%), *Candida* (3%), *Cladosporium* (2.9%), *Schizophyllum* (1.6%), *Fusarium* (1.4%), *Gleotinia* (1.3%), and *Penicillium* (1.15%). According to the core microbiome analysis, the prevalence of all the 68 genera ranged from 0 to 1 (S1 Table).

A hierarchical clustering analysis revealed that the prevalence of the genera of both bacteria and fungi varied considerably for the site, season, and different size fractions of dust (S2 Fig). The spatio-temporal variations are explained in detail in the text below.

### Occurrence of potential species

Many of the genera detected above were known to be associated with human diseases. For example the bacterial genus of *Pseudomonas* are opportunistic human invaders. We therefore looked into the possible occurrence of the associated species. Classification of the bacterial genera into species returned an overwhelming proportion of unclassified OTUs (71%) followed by an uncultured bacterium (*ca*. 26%) with a very few classified species (RA < 0.5%). Therefore we were not able to make any strong conclusions on the species level classification of the bacterial community.

However in case of fungi, a species level analysis returned 41% unassigned OTUs with the remaining classified into 89 species. Among the fungi the most dominant species was of uncultured *Cladosporium* (15%) followed by *Alternaria brassicae* (9%) and three species of *Cryptococcus*, namely *C*. *oeriensis* (6%), *C*. *albidus* (4%) and *C*. *stepposus* (0.5%). *Candida parapsilosis*, *Aspergillus gracilis*, *Aspergillus penicillioides*, *Schizophyllum commune*, and *Gloeotinia temulenta* had abundances falling in the range of 1.25–2.5% (Table 2). Similar to the genera, 78 species out of 89 (88.0% of the population) had abundances below 1.0% and were considered as other. Among the dominant types of fungi, the species of *Cladosporium*, *Candida*, and *Aspergillus* are known to be associated with allergenic reactions in humans.

**Table 2:** Predominant fungal species of dust associated microbiome of Kuwait.

Fungal Species	RA	AA
uncultured ***Cladosporium***	14.8%	80188
*Alternaria brassicae*	8.6%	46447
*Cryptococcus oeriensis*	5.6%	30610
*Cryptococcus albidus*	4.0%	21755
***Candida parapsilosis***	2.4%	13002
*Aspergillus gracilis*	2.0%	10667
***Aspergillus penicilliodes***	1.8%	9536
*Schizophyllum commune*	1.6%	9376
*Gleotina temulenta*	1.3%	8622
*Cryptococcus carnescens*	0.96%	6920
*Capnodiales sp*TR006	0.81%	5205
*Aspergillus versicolor*	0.56%	4385
*Cryptococcus stepposus*	0.51%	3060

Fungal species above 0.5% RA (Relative Abundance) AA (Actual Abundance) are enlisted; species highlighted in bold are predicted as opportunistic human pathogens.

### Spatial variations

**Fig 2:**
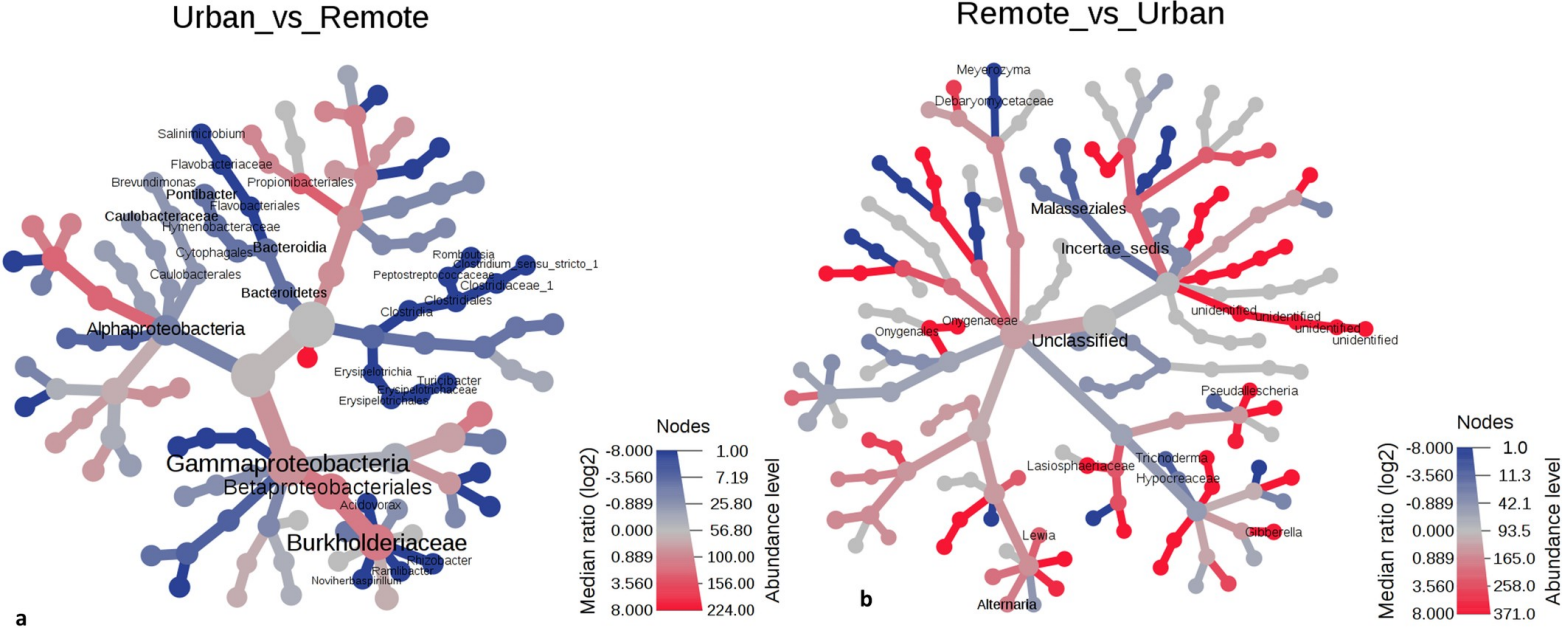
Spatial variations at various taxonomic levels presented on a differential heat tree (a) Bacterial and (b) Fungal communities. Only the significantly different taxonomic ranks are shown on the tree. (dark shade-remote population; light shade-urban communities).

Spatial variations were observed at all the taxonomic levels of bacteria between the remote and the urban sites (Fig 2A). At the phylum level, *Bacteriodetes* was completely absent at the urban site (Wilcoxon’s P = 0.0006). Among the classes, *Gammaproteobacteria* and *Alphaproteobacteria* dominated at both the sites, however, the RA were significantly variable (Wilcoxon P < 0.05). The orders *Cytophagales*, *Flavobacteriales* were only detected at the remote site. Other orders such as *Betaproteobacteriales*, *Caulobacterales*, *Propionibacteriales*, *Erysipiotrichales*, and *Clostridiales* differed in RA at both the sites (Wilcoxon’s P < 0.05). Marked differences in RA of families of *Hymenobacteraceae*, *Caulobacteraceae*, *Burkholderiaceae*, *Erysiphilotracheace*, and *Clostridiaceae* were also recorded at P < 0.05. A total of ten genera namely *Brevundimonas* (P = 0.040), *Turicibacter* (P = 0.011), *Salinimicrobium* (P = 0.028), *Clostridium* (P = 0.000), *Acidovorax* (P = 0.010), *Romboutsia* (P = 0.003), *Rhizobacter* (P = 0.035), *Pontibacter* (P = 0.002), *Ramlibacter* (P = 0.041) and *Noviherbaspirillum* (P = 0.031) were recorded to be differentially distributed among both the sites. Interestingly, a LDA at P ≤ 0.05 (owing to the limited number of tests n = 20, only the P value was considered) revealed all the above genera to be significantly differentially abundant.

In fungi, inter-site differences were less pronounced as compared to the bacterial taxonomies. No significant differences were observed at the phylum and class level (Fig 2B). At subsequent ranks, order *Onygenaceae*, *Lasisphaeriaceae*, family *Onygenales*, *Malasseziales* and genera *Alternaria*, *Lewia*, *Meyerozyma*, *Gibberella* and *Pseudallescheria* were recorded to be differentially abundant at Wilcoxsons P < 0.05. LDA analysis also yielded 7 genera to be significantly different at P ≤ 0.05 (S2d Table).

**Fig 3:**
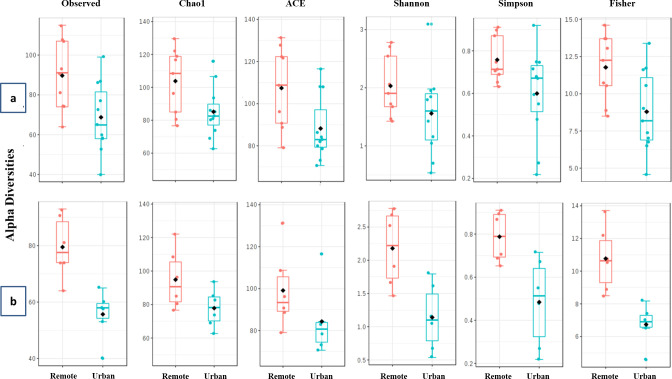
Alpha diversity plots of (a) Bacterial and (b) Fungal communities. Students T-test was employed for comparisons when the experimental factor was sample collection site. Observed alpha diversity estimates are based on unique taxa richness or counts in each sample; Chao1 and ACE diversities are based on low abundance taxa richness; Shannon, Simpson and Fisher estimates are based on both taxa evenness (distribution) as well as richness.

Our results were corroborated by the estimations of alpha diversities. These indices were always higher at the remote site compared to the urban site for both fungal (1.1–1.3 folds) and bacterial (1.2–2.1) populations (Fig 3A and 3B). A T-test conducted to compare the means of alpha diversities of the bacterial genera distributed within the site of sample collection returned significant variations in terms of genus richness (Observed, P = 0.016; Chao1, P = 0.035; ACE, P = 0.028) as well as evenness (Simpson, P = 0.041; Fisher, P = 0.010) except Shannon (P = 0.104). For the fungal species none of the T-test comparisons were significant.

**Fig 4:**
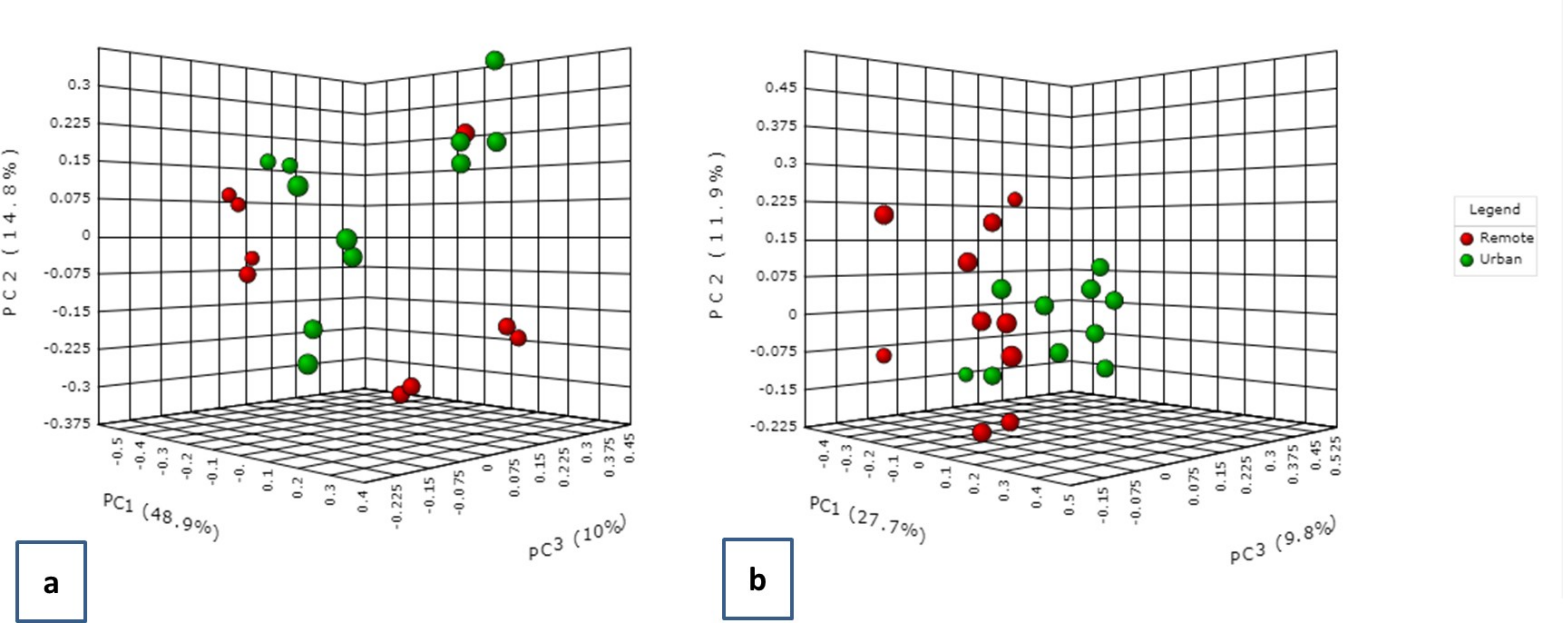
Beta diversity clustering based on PCoA on the Bray Curtis distances of (a) bacterial genus and (b) fungal genus distributed between two locations in Kuwait. The two colours represent the two sites Abdally/remote and Kuwait city/urban.

The community structure of microbial populations was predicted through the PCoA. Both bacterial and fungal communities existed as two partially overlapping clusters (Fig 4A and 4B). The statistical methods of PERMANOVA and ANOSIM were used to test the strength of the sample grouping. An R-value closer to 1 means the groups are dissimilar, while an R-value near 0 means negligible dissimilarity. In our case both the tests returned an R^2^ of 0.18 (P < 0.004) and 0.306 (P < 0.004) suggesting low to moderate diversity among the bacterial communities in all the samples with respect to distribution between sites. Very low but significant PERMANOVA R^2^ (0.096; P < 0.021) and ANOSIM R^2^ (0.153; P < 0.014) were recorded for fungal genera as well. As mentioned previously, values approaching 1 are highly different; however, considering the geographic proximity of remote and urban sites in Kuwait, these values appear to be reasonable as a measure of high diversity for microbial populations.

### Seasonal variations among the air microbiome

**Fig 5:**
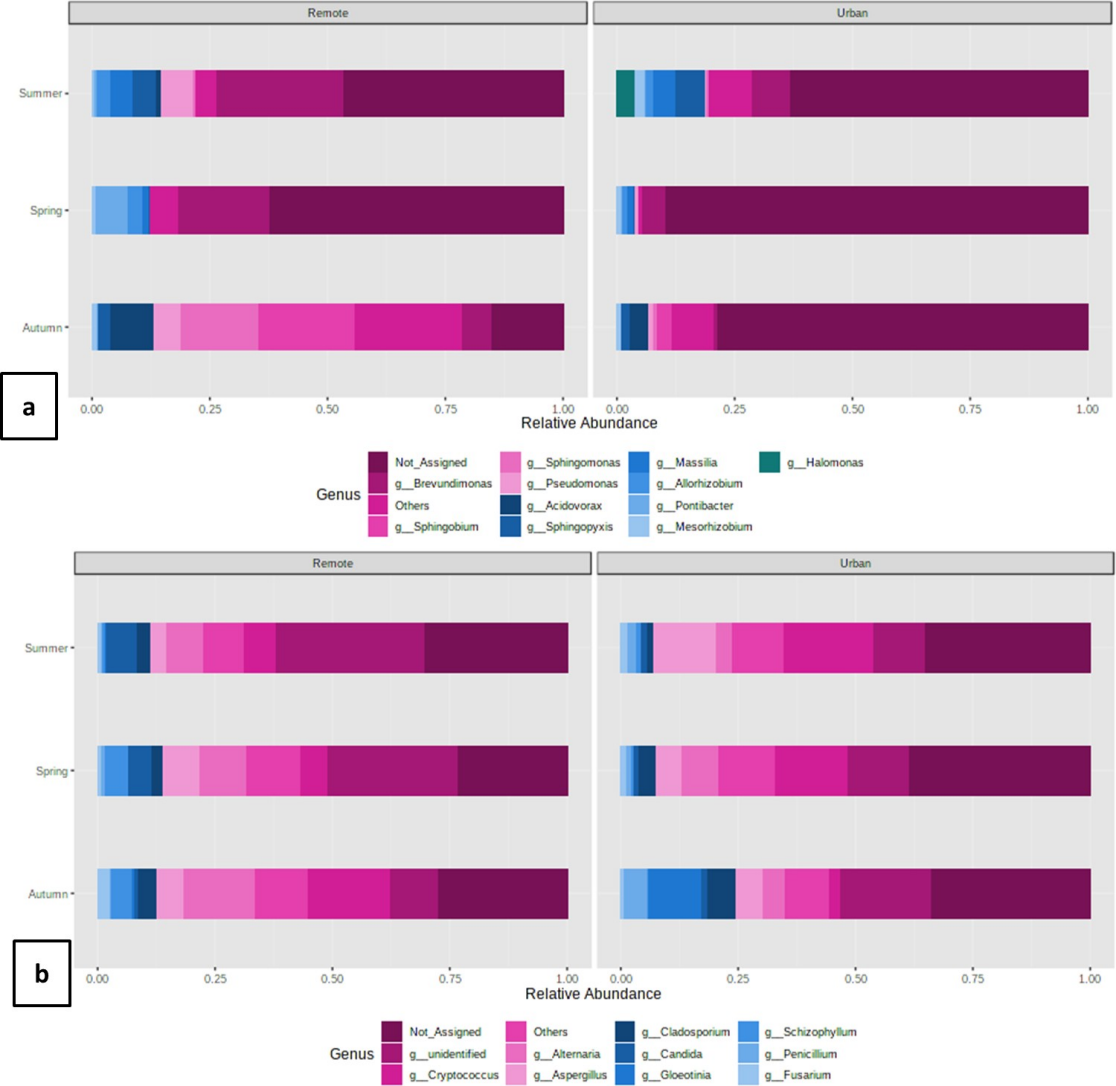
Bar plots representing the seasonal variations in relative abundances of predominant genus of (a) Bacteria and (b) Fungi. The taxa above 100 OTU counts are represented on the plots. The rest are binned together as others.

Similar to the spatial variations, differences were also observed among the bacterial populations in three different seasons of sampling. As seasonality was determined to be the driving force in both healthy and diseased states of the population, and the genus was found to be the smallest reliable taxonomic unit, only differences in genus were studied in more detail. While analyzing the genera we noticed that the predominant category (RA > 1%) was varied in terms of their RA (S3a Table). Considering the fact that these pre-dominant categories are ones representing the core microbiome, and any differences in their RA would alter the physiological state of the whole consortia, we studied their variations in more depth. In spring and summer, *Brevundimonas* dominated both at the remote site (mean = 0.24; 0.28 respectively) as well as at urban site (mean = 0.05; 0.09 respectively). The autumn season was more variable as two different genera prevailed at the remote site (*Sphingobium-* mean = 0.20) and urban site (*Acidovorax-* mean = 0.04). It was also observed that in most of the cases, the RA of the same genus at the remote site was relatively higher than at the urban site. Our results were indicative of the prevalence of specific genera in particular months at a particular site (Fig 5A). It is important to note here that the Other category (RA < 1%) also contributed considerably to the overall diversity (Autumn-Remote-0.23; Autumn-Urban- 0.08; Spring-Remote-0.08; Spring-Urban-0.01; Summer-Remote- 0.05; Summer-Urban-0.11). However, it was not possible to analyze each genus individually.

Like the bacterial genera, we also analyzed the seasonal diversity in the fungal microbiome (S3b Table). Seasonal variations were observed at all taxonomic positions (Wilcoxson p < 0.05). The RA of the predominant genera also varied depending upon the season of collection. *Alternaria* dominated in spring (0.10) and summer (0.08) at the remote location whereas *Cryptococcus* (0.18) was more in autumn. A completely different trend was recorded at the urban site where the latter genus was highly prevalent in spring (0.16) and summer (0.19), whereas *Gleotina* (0.11) dominated during autumn (Fig 5B). In the case of fungal genera, also an overwhelming proportion (Autumn-Remote-0.28; Autumn-Urban- 0.34; Spring-Remote-0.22; Spring-Urban-0.39; Summer-Remote- 0.29; Summer-Urban-0.34) of not assigned (NA) genera were recorded in all the seasons at both the sites suggesting the probable presence of novel forms. The other genera contributed 0.08–0.11 (Autumn-Remote-0.10; Autumn-Urban- 0.09; Spring-Remote-0.11; Spring-Urban-0.11; Summer-Remote- 0.08; Summer-Urban-0.10).

**Fig 6:**
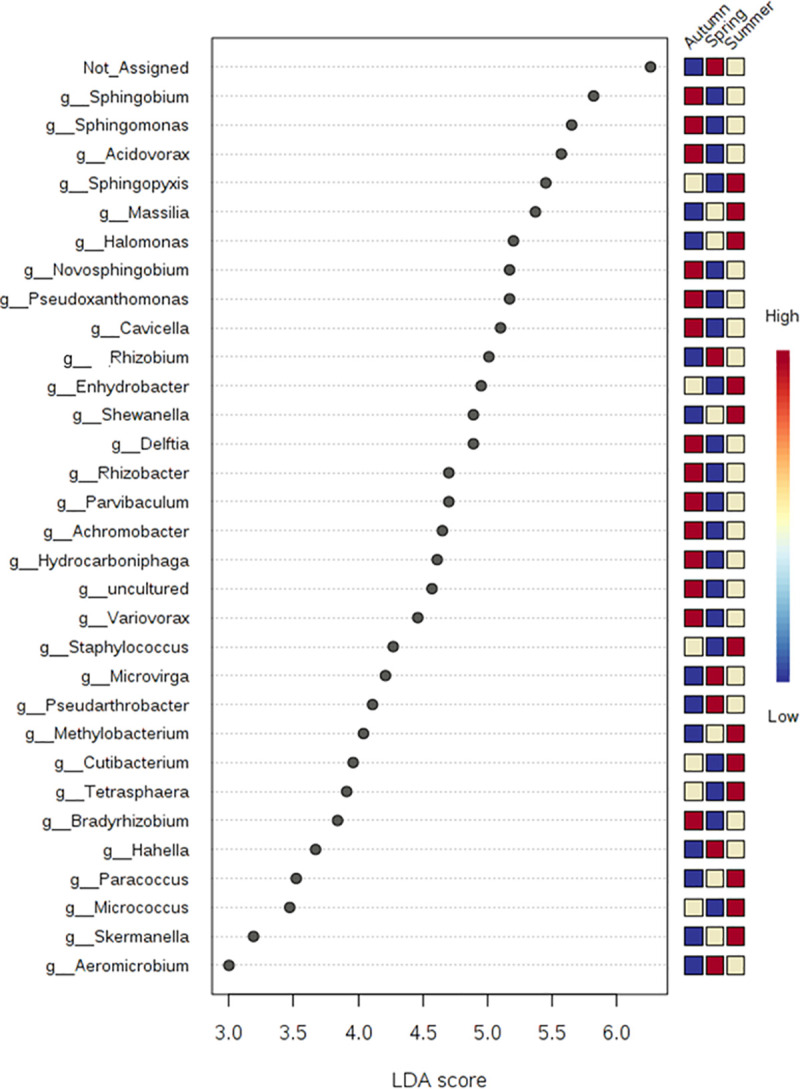
LDA analysis based on non-parametric factorial Kruskal-Wallis (KW) sum-rank test of bacterial genus among three seasons (p & q ≤ 0.05). The heat map on the right side of the figure explains the abundance of the genera in three different seasons depicted by the three colours. Blue (low abundance), beige (median abundance) and red (high abundance).

A rich set of 32 bacterial genera were found to be significantly differentially abundant after the LDA analysis (P ≤ 0.05). Among this set of genera, five genera belonged to the predominant category. The LDA score ranged from 2.81 to 6.26 with a maximum of 5.5 for *Sphingobium* and a minimum for *Aeromicrobium* (Fig 6). A total of 6 fungal genera were also detected as differentially abundant at P ≤ 0.05 (S2e Table). None of these genera belonged to the pre-dominant category indicating that the effect of seasonality is more pronounced on the other category rather than the dominant fungal forms.

**Fig 7:**
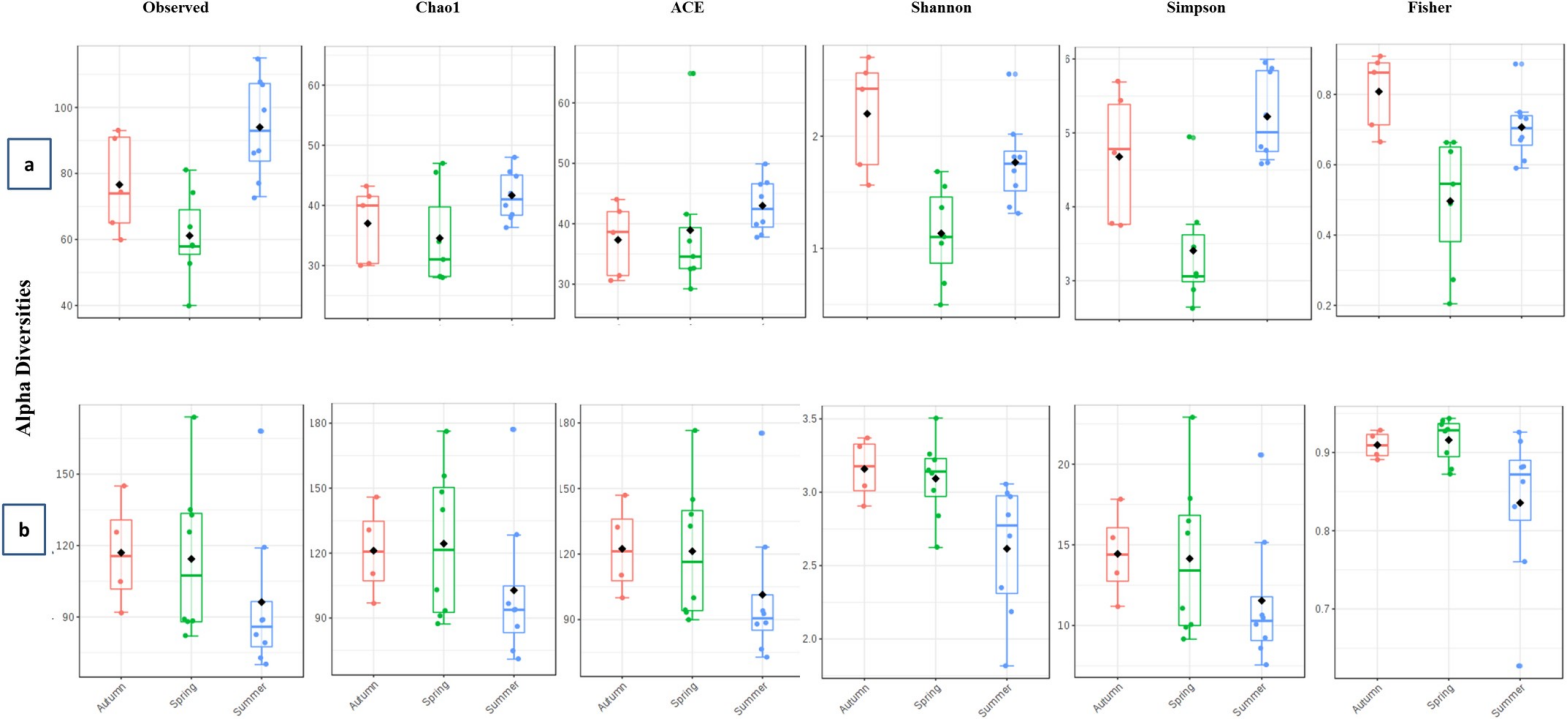
Season wise alpha diversity plots of (a) bacterial and (b) fungal communities. All comparisons were made through analysis of variance (ANOVA). Observed alpha diversity estimates are based on unique taxon richness or counts in each sample; Chao1 and ACE diversities are based on low abundance taxon richness; Shannon, Simpson and Fisher estimates are based on both taxon evenness (distribution) as well as richness.

We compared the indices of alpha diversity within bacterial populations in three different seasons and found significant variations at (P = 0.001; 0.002; 0.003 and 0.000 for Observed, Shannon, Simpson, and Fisher, respectively) except for Chao1 (P = 0.121) and ACE (P = 0.451). Observed, Chao1, ACE and Fisher values were maximum in the summer season. It is likely that the prevailing conditions supported the growth of numerous species resulting in a richly diverse bacterial population (Fig 7A). The levels of Shannon and Simpson were highest in autumn suggesting that post multiplication, the species balance themselves in the consortia pertaining to the prevailing environmental conditions.

Fungal genus counts and evenness varied with respect to the season of sample collection (Fig 7B). Although a higher number of species were recorded in autumn, the comparisons of alpha diversity indices exhibited no significance at a 95% confidence interval (P ≥ 0.05). These numbers were comparable with those detected in spring. Summer season exhibited the lowest species abundances suggesting that higher temperatures are less likely to promote fungal growth and propagation.

**Fig 8:**
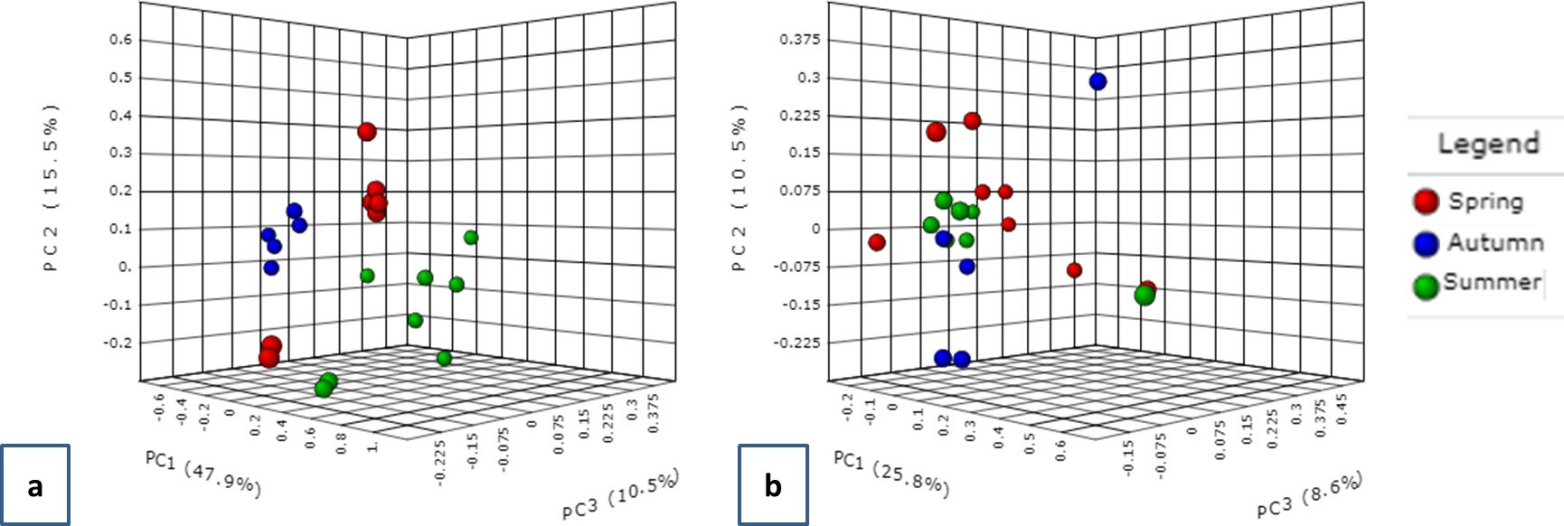
Beta diversity clustering based on PCoA on the Bray Curtis distances of (a) bacterial and (b) fungal genus distributed during the three seasons in Kuwait. The colours represent the three seasons blue (autumn); red (summer); green (spring).

The beta diversity analysis distributed the samples into three distinct clusters according to seasonality (Fig 8A and 8B). It was evident from the PCoA plots that the bacterial communities were distantly apart in all the three seasons. This was also reflected by their high PERMANOVA (R^2^ = 0.539; P-value < 0.001) and ANOSIM coefficients (R^2^ = 0.757; P < 0.001) (Fig 8A). For fungal populations, three clusters were observed with autumn communities being in partial overlap with the spring group. The summer and spring populations appeared as overlapping clusters on the PCoA plot (Fig 8B). The PERMANOVA and ANOSIM coefficients for fungal beta diversities were recorded as 0.134 (P <0.112) and 0.005 (P <0.441), respectively.

### Differential genera associated with different size fractions of dust

In the present investigation, the dust fractions were collected over six stages (Stage 1–5 respirable; stage 7-inhalable) in a particular season at the respective sites (Table 1). The air particulate matter in each size fraction was between the aerodynamic diameter (Dp) sizes ranges of < 0.39 (backup high volume filter-Stage 7), 0.39 to 0.69 (Stage 6), 0.69 to 1.3 (Stage 5), 1.3 to 2.1 (Stage 4), 2.1 to 4.2 (Stage 3), 4.2 to 10.2 (Stage 2) and > 10.2 μm (Stage 1). During sample collection, the stage 6 filters were disrupted and therefore the microbial content of this stage could not be investigated. Stage 1–5 is the larger size fraction of dust that is inhaled but resides in the upper respiratory tract, whereas the stage 7 fractions are finer and make their way in the pulmonary region where the air exchange takes place. The latter is more hazardous and prone to cause diseases. The presence of unidentified DNA in all the size fractions suggested the occurrence of biological material like viruses or pollen besides the already identified bacteria and fungi. The bacterial and fungal populations in the size fractions from which quantifiable DNA was obtained but did not produce any amplification can be considered as below detection limits.

**Fig 9:**
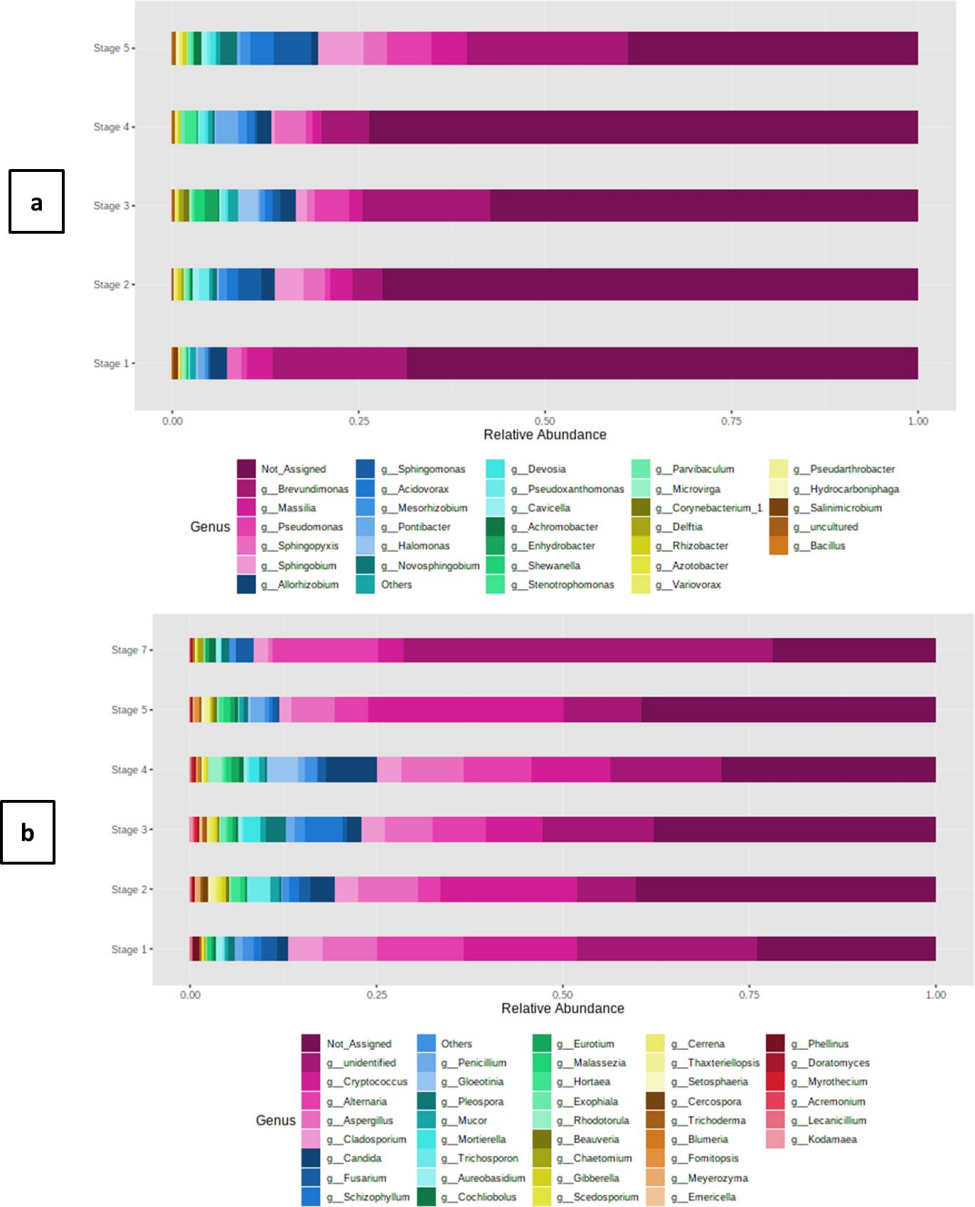
Relative abundance of (a) bacterial and (b) fungal genus associated with different size fractions of dust at the remote and urban sites. Bacterial genera were only found in the respirable fractions (stage 1–5). Fungal genera were detected both in respirable (stage 1–5) as well as in the inhalable (stage 7) size fraction of dust.

In our study bacterial genera found were associated with only respirable fractions *i*.*e*. stages 1–5. None of the inhalable (stage 7) dust fractions appeared positive for bacterial communities. All the genera associated with the different size of dust fractions varied in their relative abundances (Fig 9A). The alpha diversity and beta diversity analysis demonstrated the differences to be non-significant. In addition, none of the bacterial genera were found to be differentially abundant through the LDA analysis.

**Fig 10:**
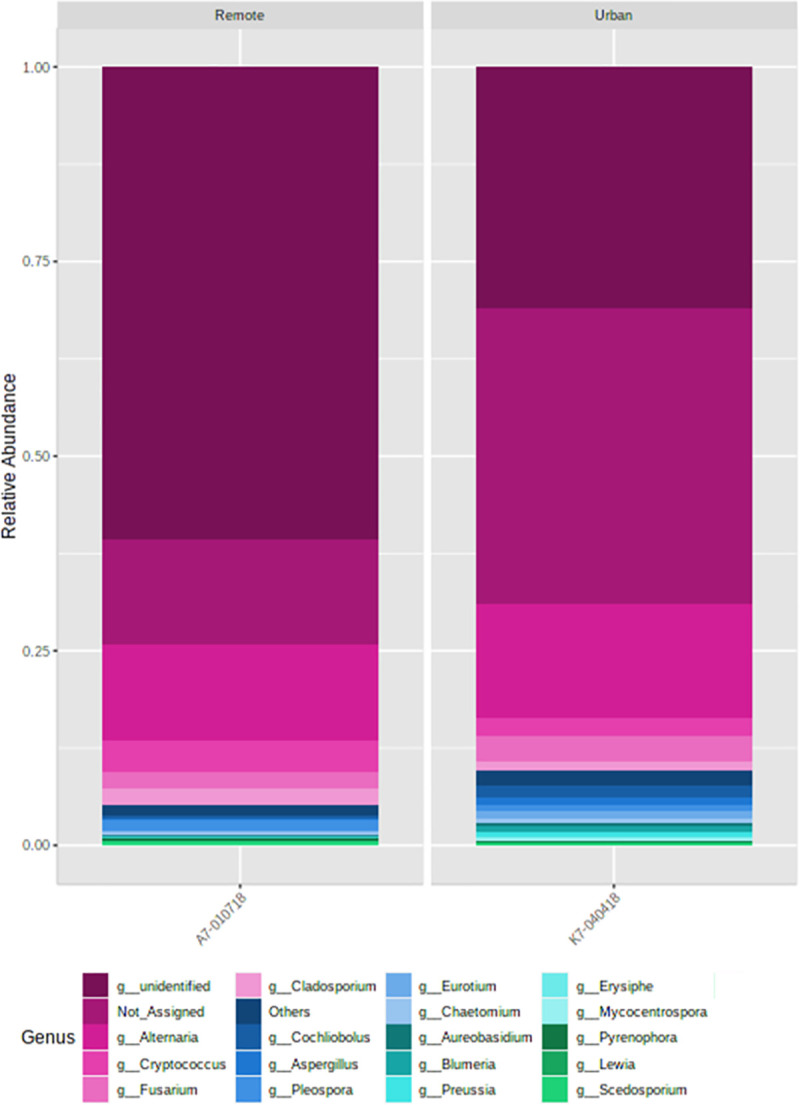
Fungal species associated with the inhalable size fractions of the dust at the (a) remote and (b) urban sites. Relative abundances were recorded to be higher at the remote location as compared to the urban place.

The fungal genera distribution also followed a similar pattern (Fig 9B). The LDA analysis revealed three fungal genera (P ≤ 0.05) to be significantly different among different size fractions of dust (S2f Table). We also examined the fungal taxa associated with the inhalable size fraction of dust, and recorded ~ 60 and 100 classified genera and species, respectively (S4 Table). Predominance of *Cladosporium*, *Alternaria* and *Aspergillus* and their species were detected in the inhalable fractions at both the sites (Fig 10).

**Fig 11:**
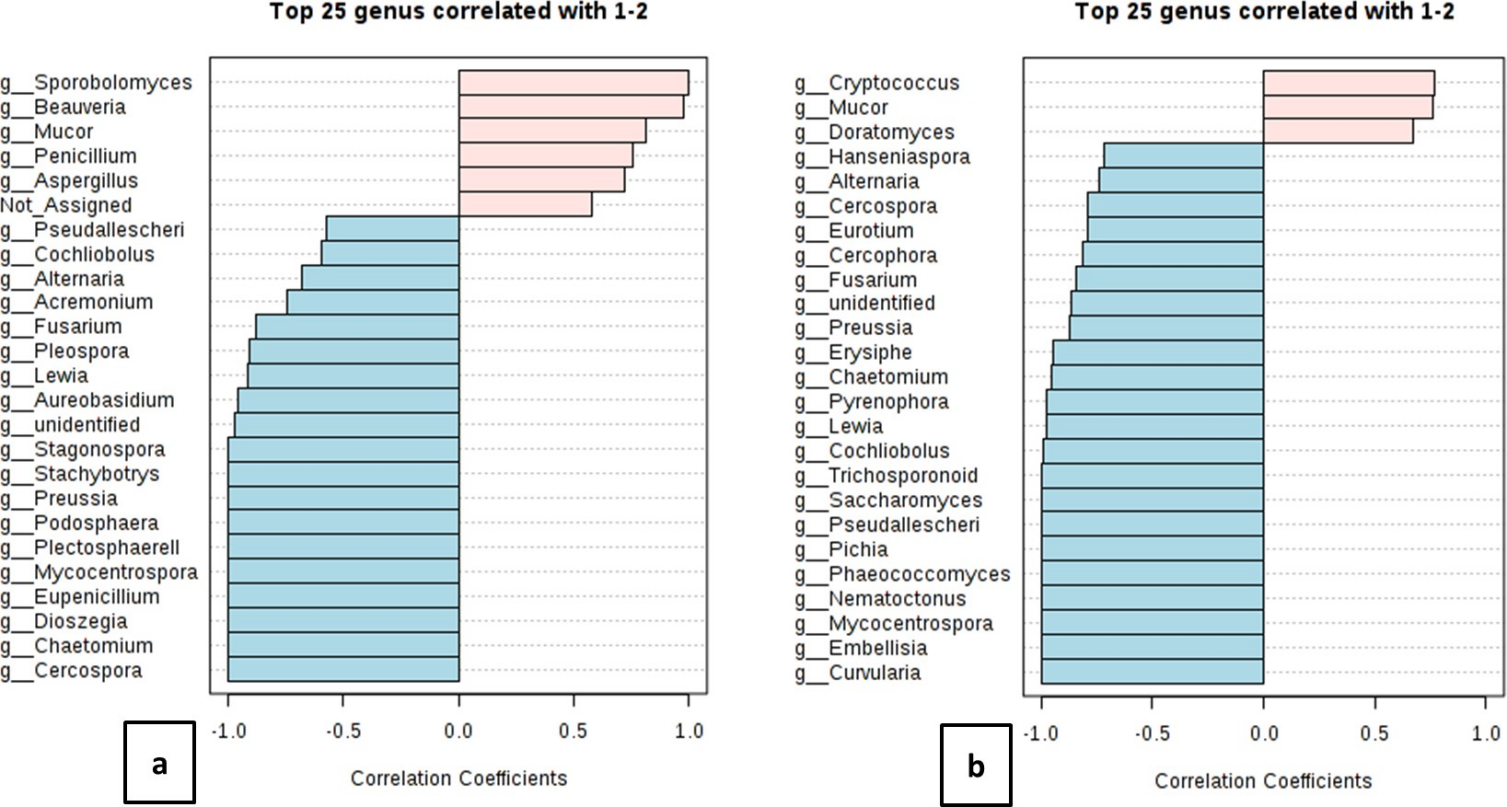
Correlation analysis of inhalable (a) A7-010718 and (b) K7-040418 fractions with their respirable counterparts. The top twenty five fungal genera with significant correlations coefficients are represented in the bar plots. 1 corresponds to inhalable whereas 2 corresponds to the respirable fractions.

We also performed a correlation analysis among the genera associated with both the inhalable and respirable fractions particularly for these months. We discovered n = 6 and n = 3 genera correlated (r^2^ ≥ 0.5) between both the fractions at the remote and urban site, respectively (Fig 11A and 11B). This suggests that the correlations of fungal genera between different size fractions of dust contribute towards the existence and absence of specific fungal genera. To some extent this would also affect the overall consortia. Moreover, investigations on the interactive dynamics (both positive and negative) among these taxa would be crucial to further understand the microbial dysbioses with regards to health perspectives associated with different dust fractions. Furthermore, increasing the sampling frequency would help shed more light on the role of size fractions on fungal community composition.

## Discussion

As a first consideration, this study revealed a major conserved contingent of bacteria and fungi representative of a local-air microbiome which appears to characterize the Kuwaiti pool of atmospheric microbes that occur in both remote and urban sampling stations of the country irrespective of the wind direction and temperature regime. This wide group of bacterial and fungal genera recurrently exists in spite of site and temperature conditions, and thus represents the global Kuwaiti air microbiome.

### Common air microbiome due to the common origin of air mass

The taxonomic distribution followed a characteristic pattern of air inhabiting bacterial and fungal populations. Our results were corroborated by other studies wherein *Proteobacteria* were the predominant bacterial phyla in the airborne dust samples followed by *Firmicutes*, *Actinobacteria*, and *Bacteroidetes* [28–30]. Reports have also shown that airborne bacteria represent diverse groups with the most abundant bacteria from the classes *Bacilli*, *Clostridia*, *Alphaproteobacteria*, *Betaproteobacteria*, and *Gammaproteobacteria* as found in the current investigation [31]. However, further down considerable variations at orders, families, and genera were found compared to other studies. For instance, the abundance of *Bacillales*, *Salinisphaerales* and *Enterobacteriales*, *Clostridiales* and *Saprospirales* were recorded in air above the Eastern Mediterranean sea as compared to *Pseudomonadales*, *Caulobacterales*, *Sphingomonadales*, *Rhizobiales*, *Oceanospirillales*, and *Xanthomonadales* in the present scenario [31]. Cross study comparisons of bacterial populations are difficult owing to variations in sampling techniques, laboratory protocols, bacterial primer selection, sequencing methods, and data analysis pipelines. The predominant bacterial genera were *Brevundimonas* (12%), *Sphingobium* (3.3%), *Sphingopyxis* (2.7%), *Pseudomonas* (2.5%), *Sphingomonas* (2.4%), *Massilia* (2.3%), *Acidovorax* (2.0%), *Allorhizobium* (1.8%), *Halomonas* (1.3%), and *Mesorhizobium* (1.1%). Similar ubiquitous genera dominated by *Sphingobium* were identified in the core airborne microbiome in Spain [32]. The most common bacterial species of *Bacillus*, *Staphylococcus* and *Ps*. *aeruginosa* were identified from the dust fallouts in a study in Saudi Arabia [33].

Among the fungal taxa, *Basidiomycota* and *Ascomycota* were the most frequently detected fungal phyla as reported in other studies also [32]. Investigations carried out on the composition of fungal spores in Jordan [34] highlighted that the dominant fungal collections in the arid climate were *Cladosporium* (29.1%), *Fusarium* (20%), *Alternaria* (7.7%), *Ulocladium* (6.5%), *Penicillium* (4.2%) and *Aspergillus* (3.6%). They indicated that these genera were also dominant in the air of Kuwait, Saudi Arabia, Qatar and the eastern desert of Egypt. Al Awadhi [35] also recorded the presence of two types of fungi *Aspergillus* spp and *Alternaria* spp. in dust samples from Kuwait. Studies from Riyadh, Saudi Arabia [33] reported *Aspergillus* sp. as the most common fungus. We reconfirm the occurrence of these genera in our core microbiome on account of geographic proximity of Jordan and Saudi Arabia with Kuwait. The genera of *Aspergillus*, *Penicillium*, *Candida*, and *Cryptococcus* were also identified as common skin commensals in Chinese individuals [36].

### Health implications of air microbiome of Kuwait

The taxa observed in the present study are those most commonly found in residential housing as well as schools and other indoor environments [28, 29]. Furthermore, they are also the chief components of the human skin [37] and nasal microbiota [38] indicating the role of these microorganisms in human health. It has been known for centuries that these ubiquitous microorganisms in the atmosphere, are capable of long-distance dispersal and often inhabit the human skin where these airborne bacteria and fungi can have myriad effects on human health [39]. The species of many bacterial genera (*Achromobacter*, *Pseudomonas*, *Corynebacterium*, *Agrobacterium*, *Shewanella* etc.) identified in our study act as opportunistic pathogens. On a slightly different note, Leski [40] described a high prevalence of human pathogens including *Mycobacterium*, *Brucella*, *Clostridium perfringens*, *Bacillus* and *Coxiella burnetii*, a highly infectious potential bio-warfare agent in samples of fine topsoil particles and airborne dust collected in 19 locations in Iraq and Kuwait.

With respect to fungi we recorded a high prevalence of *Cladosporium* [41], *A*. *penicillioides* [42] and *Candida parapsilosis* [43] that are known to be associated with allergic rhinitis and human pathogenicity. Al Awadhi [35] also recorded the presence of two types of fungi, *Aspergillus* spp and *Alternaria* spp., in dust samples from Kuwait. Studies have shown that fungi in indoor air are dominated by those from outdoor air [39, 44]. Certain species of fungi are associated with human skin [45] and may be released as bioaerosols upon shedding. Yamamoto et al. [46] found that floor dust in classrooms was enriched in skin-associated yeasts, such as the genera *Rhodotorula*, *Candida*, *Cryptococcus*, *Malassezia*, and *Trichosporon*.

Although, the RA of these species were minimal as compared to overwhelming concentrations of unclassified/unculturable forms, however, their mutual interaction raises a concern and provides a lead towards further research to be undertaken at the functional level, as it is the bacterial/fungal species that interact with the host to establish a beneficial, commensal or pathogenic relationship [47]. In addition our assumptions are based on 16s profiling algorithms that do not provide efficient taxonomic resolution and accuracy to perform species-level associative studies. Therefore, whole genome metagenomics should be carried out to get accurate identification up to species level, and information on other coexisting organisms such as archaea, viruses and eukaryotes needs to be obtained.

### Species richness, evenness and diversity attributed to spatio-temporal variations

Microbes in the air are predominantly associated with particles, hence when there are more particles in the air, there is likely to be a larger microbial load or microbial diversity [47]. In our study we demonstrated that the Observed, Chao1, ACE, Shannon, Simpson and Fisher indices were higher in the remote site as compared to the urban location. The air mass is supposed to enter Kuwait from Abdally (remote) region with more particulate matter and therefore the bacterial and fungal diversity are expected to be higher in this region. More unique OTUs and a higher microbial load were detected in oceanic air samples when more mineral dust particles were observed [31]. High abundance rates of bacterial communities were recorded at dust events registering higher dust particles in the Gobi desert [48].

Moreover, urbanization leads to homogenization of the airborne microbiota, with more urban communities exhibiting less continental-scale geographic variability than in more rural areas [39]. In the present investigation, the Abdally region is less populated lying on the outskirts and therefore resembles a rural area, whereas KISR is near the city center with dense urban populations. Our results are in agreement with some of the other reports where higher bacterial/fungal diversities were detected in the rural areas compared to the urbanized locations [49–51].

The temperature has also been acknowledged as a principal factor influencing bacterial community diversity and function, which directly reflects seasonal changes. Our samples were collected over three different seasons. The hierarchial clustering on cumulative samples within a season revealed significant variations. These results were corroborated by the observations of the observed OTUs which recorded the highest for bacterial and lowest for fungal genera. This can be attributed to the fact that the summer season promotes the multiplication of bacteria whereas it is less conducive for fungal growth [52]. Similarly, spring and autumn probably being the periods of moderate temperatures regimes, favors fungal growth, but are less preferred by the bacterial populations and hence less diverse populations [53]. Our findings also suggest the temperature regimes in spring and autumn support the growth of cryptic fungi. Additional investigations through advanced approaches of whole-genome metagenomics would provide better insights into the identification of these novel fungal genera. Studies from Riyadh, Saudi Arabia [33] reported higher microorganisms counts in spring and summers and lower ones in autumn and winters during 2012, and attributed this to increased incidences of dust storms. We inferred that the passage of time strongly affected the distribution and richness of bacterial and fungal populations [52]. These differences in temporal dynamics cannot be fully explained at this point, and need to be further investigated by increasing the sampling time points, including assessments of other associated microbial communities.

### Community structure defined by the socio-economic and environmental conditions

Many previous studies showed seasonality is a factor controlling airborne bacterial emissions and community composition [52, 54–57]. In agreement with the above, our results reveal a diverse population of bacteria in Kuwaiti airborne dust that changed over time and between locations. Temporal variations supported by ANOSIM analyses on unweighted UniFrac distances clustered the phyla into Pacific and continental sources [58]. It is also expected that urban and suburban communities may harbor different outdoor bacteria, reflecting differences in land use and population density, etc [59]. Kuwait is a small country with a land area of 6,880 sq mi (17,818km^2^). The remote area is sparsely populated with the majority of the human population concentrated in the urban area. The distance between the two locations is a bare minimum of 100 km^2^. Although the land distance is small between the two sites of sample collection, the work atmosphere is entirely different. The remote area represents the agricultural land with much farming activity being conducted and it is sparsely populated. In contrast, the urban area is densely populated, where people are found working in offices and with much automobile traffic in abundance. We attribute the differences in the bacterial communities among both the sites to these factors. The small land area without any demographic barriers would be the reason behind the partially intersecting bacterial populations. Therefore, in the present investigation overlapping clusters were recorded for the bacterial genera among the remote and urban sites.

However, the fungi of the airborne dust did not change over time and showed very limited variations between locations. Unlike bacterial communities, fungal populations remained relatively stable between sampling regions and seasons. These findings are contrary to our hypothesis as both time and region exert a significant impact on the fungal community structure as reported in other air microbiomes [44]. However, influential factors on a continental scale (e.g., temperature or precipitation) [30, 60] are rather uniform on a regional scale, and factors that affect a rural area may not be significant for the surrounding area. The limited distance between the two sampling sites added to this effect. We therefore hypothesize that the constant presence of fungi in all the dust fractions to be the reason behind higher incidences of allergies in Kuwait, particularly in a pediatric population, when compared with European countries [11, 61] as they lack the incidences of dust storms.

### Differential abundance in different size-fractions of dust

The bacterial and fungal taxa differed in their RA with different size fractions (respirable-stage1-5 and inhalable-stage 7). Another study in agreement with our results was reported in China whereby the airborne bacterial community composition was significantly affected by PM fractions [62]. However, the size of the PM fraction did not significantly affect the diversity and richness of airborne bacterial communities as reported in other studies [62]. We reported a reasonable number of taxa to be differentially abundant between different size-fractions. From a health perspective, a more detailed examination of network dynamics between these taxa should be undertaken. It would also be interesting to study the viability status of these taxa, as metabolically active microorganisms are the potential producers of allergenic endotoxins and mycotoxins [63–65].

## Conclusions

This baseline study demonstrates the occurrence of a persistent microbial population in the size-fractionated aerosol of Kuwait. There is a spatio-temporal variation in the composition of bacterial and fungal communities. Species richness and evenness were higher in the remote site as compared to the urban site. Bacterial taxa exhibited a community structure with respect to seasonality and location, whereas the fungal distribution was more or less constant throughout the seasons and locations. The presence of fungi and bacteria in the inhalable fractions raises a potential health concern. Future studies on their metabolic status and functional dynamics would provide a deeper insight from a public health and wellbeing point of view. In addition, gaining knowledge on other communities that interact as a whole microbial consortium would also enhance our understanding of their roles in dysbiosis.

## Supporting information

S1 Fig (a) Annual windrose–Kuwait (Source: http://www.Windfinder.com/windstatistics/kuwait_city); (b) Sites of sampling (map source https://www.graphicmaps.com/kuwait) Al Abdally-Remote; Kuwait City-Urban (c) Dominant wind direction (Abdalli October 8, 2017).
(PDF)

S2 Fig Hierarchial clustering of (a) Bacterial (b) Fungal Euclidean distances employing the Ward algorithm.
(PDF)

S1 Table Taxonomy and core microbiome analysis (S1a) Bacterial taxonomic classification at phylum level; (S1b) Bacterial taxonomic classification at class level; (S1c) Bacterial taxonomic classification at order level; (S1d) Bacterial taxonomic classification at family level; (S1e) Bacterial taxonomic classification at genus level; (S1f) Core bacteriome; (S1g) Fungal taxonomic classification at phylum level; (S1h) Fungal taxonomic classification at class level; (S1i) Fungal taxonomic classification at order level; (S1j) Fungal taxonomic classification at family level; (S1k) Fungal taxonomic classification at genus level; (S1l) Fungal taxonomic classification at species level; (S1m) Core fungal biome.
(PDF)

S2 Table Linear discriminant analysis (S2a) LDA analysis of bacterial genus according to the site; (S2b); LDA analysis of bacterial genus according to the season; (S2c) LDA analysis of bacterial genus according to the stage; (S2d**)** LDA analysis of fungal genus according to the site; (S2e) LDA analysis of fungal genus according to the season; (S2f) LDA analysis of fungal genus according to the stage.
(PDF)

S3 Table Seasonal diversity in relative abundances (a) Seasonal diversity of predominant bacterial genera; (b) Seasonal diversity of predominant fungal genera.
(PDF)

S4 Table Fungal genera and species acssociated with the inhalable fractions of dust. (XLSX)

S1 Appendix Raw sequence quality and filtering parameters for OTU picking. S1a Table. Basic sequencing statistics and QIIME cut-off; S1b Table: Default parameters set in MicroBiomeAnalyst; S1a Fig: Library size overview of (i) Bacterial (ii) Fungal sequences; S1b Fig: Data rarefaction curves of (i) Bacterial and (ii) Fungal sequences.
(PDF)

S1 File Bacterial biome file generated by QIIME. (BIOM)

S2 File Fungal biome file generated by QIIME. (BIOM)
