# Supplementary material for: Spatio-temporal variations in bacterial and fungal community associated with dust aerosol in Kuwait
Source: PLoS One. 2020 Nov 5;15(11):e0241283. doi: 10.1371/journal.pone.0241283 (PMC7644028; doi:10.1371/journal.pone.0241283)
Supplement: S1 Fig — (a) Annual windrose–Kuwait (Source: http://www.Windfinder.com/windstatistics/kuwait_city); (b) Sites of sampling (map source https://www.graphicmaps.com/kuwait) Al Abdally-Remote; Kuwait City-Urban (c) Dominant wind direction (Abdalli October 8, 2017). (PDF) [file pone.0241283.s001.pdf]

**Supplementary File S1**

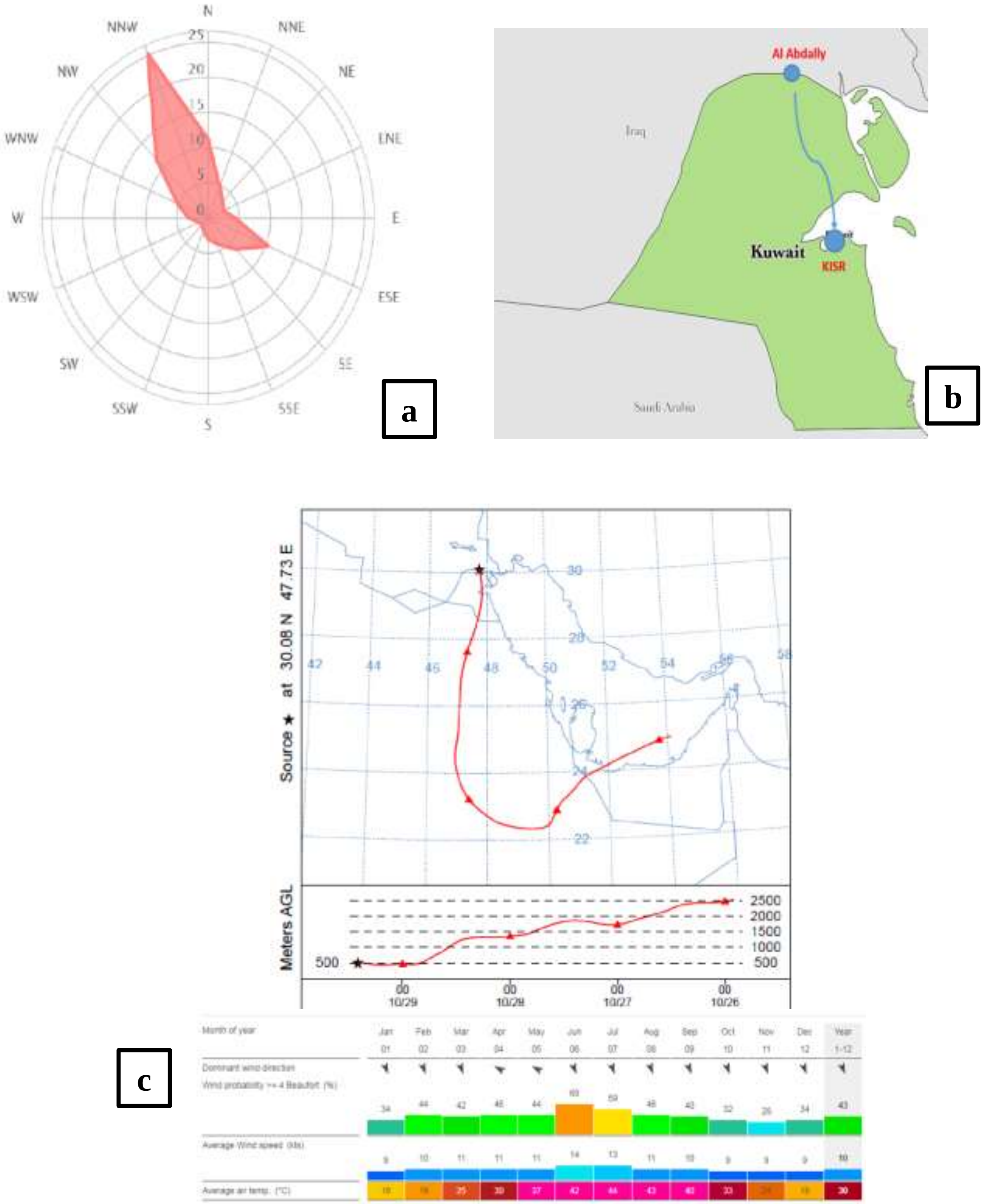


**Figure S1: (a) Annual windrose – Kuwait (Source: /kuwait_cityhttp://www.Windfinder.com / windstatistics); (b) Sites of sampling (map source https://www.graphicmaps.com/kuwait )Al Abdally-Remote;Kuwait City-Urban (c) Dominant wind direction (**Abdalli October 8, 2017).
