# Supplementary material for: Spatio-temporal variations in bacterial and fungal community associated with dust aerosol in Kuwait
Source: PLoS One. 2020 Nov 5;15(11):e0241283. doi: 10.1371/journal.pone.0241283 (PMC7644028; doi:10.1371/journal.pone.0241283)
Supplement: S2 Fig — Hierarchial clustering of (a) Bacterial (b) Fungal Euclidean distances employing the Ward algorithm. (PDF) [file pone.0241283.s002.pdf]

Supplementary File S6

Hierarchical Clustering

We corroborated our observations on taxonomic variations by the hierarchical clustering based on Euclidean distances employing the Ward algorithm. We noticed that the prevalence of all the 50 bacterial and 68 fungal genera differed with respect to the site and season.

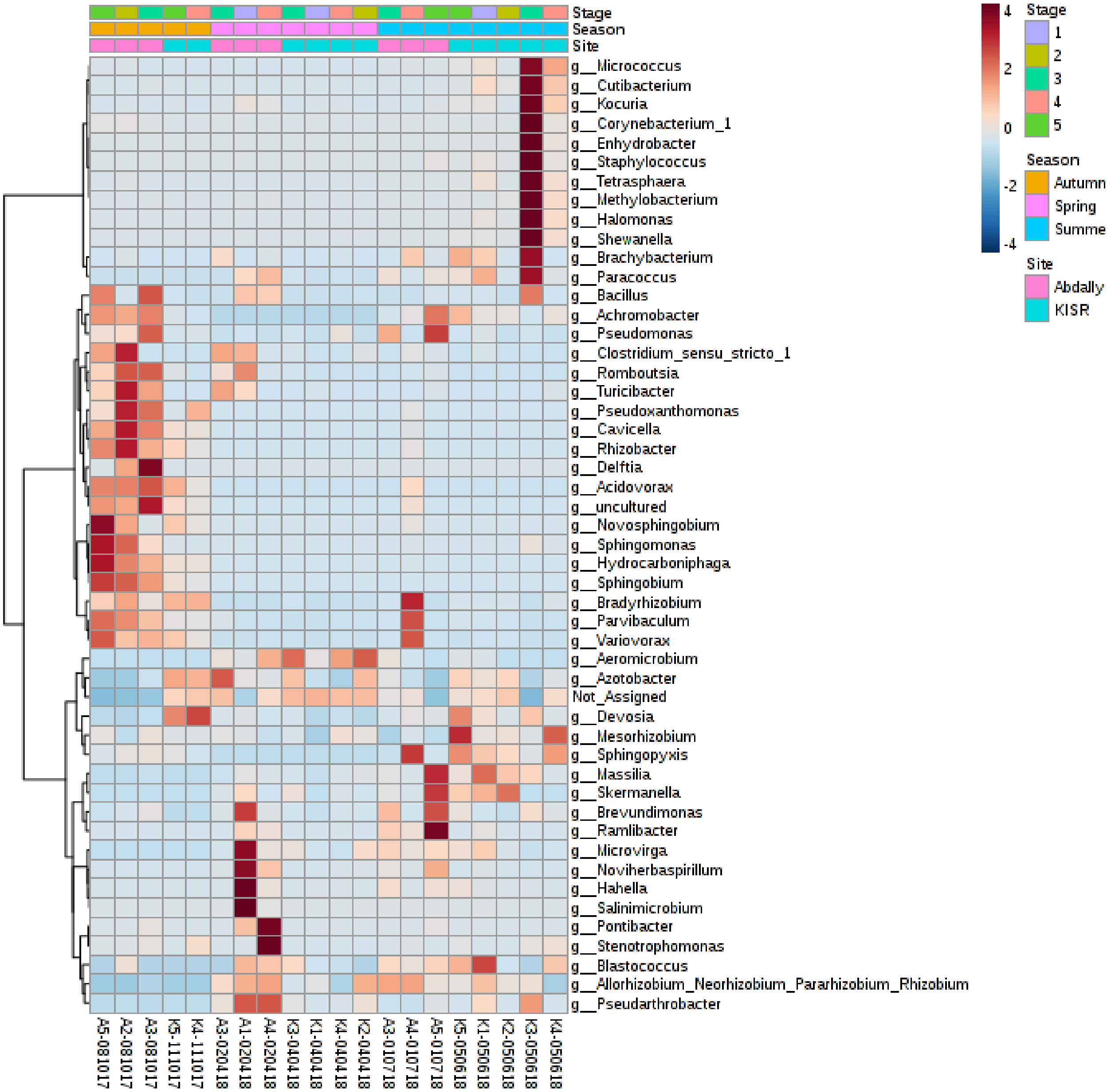

Figure S6a: Hierarchical clustering of bacterial genera. Ward Algorithm was applied on the Euclidean distances to present the spatio-temporal variations with respect to the prevalence of all the detected bacterial genera. Dark coloured boxes represent the high prevalence of a particular genus in a particular season at the specific site.

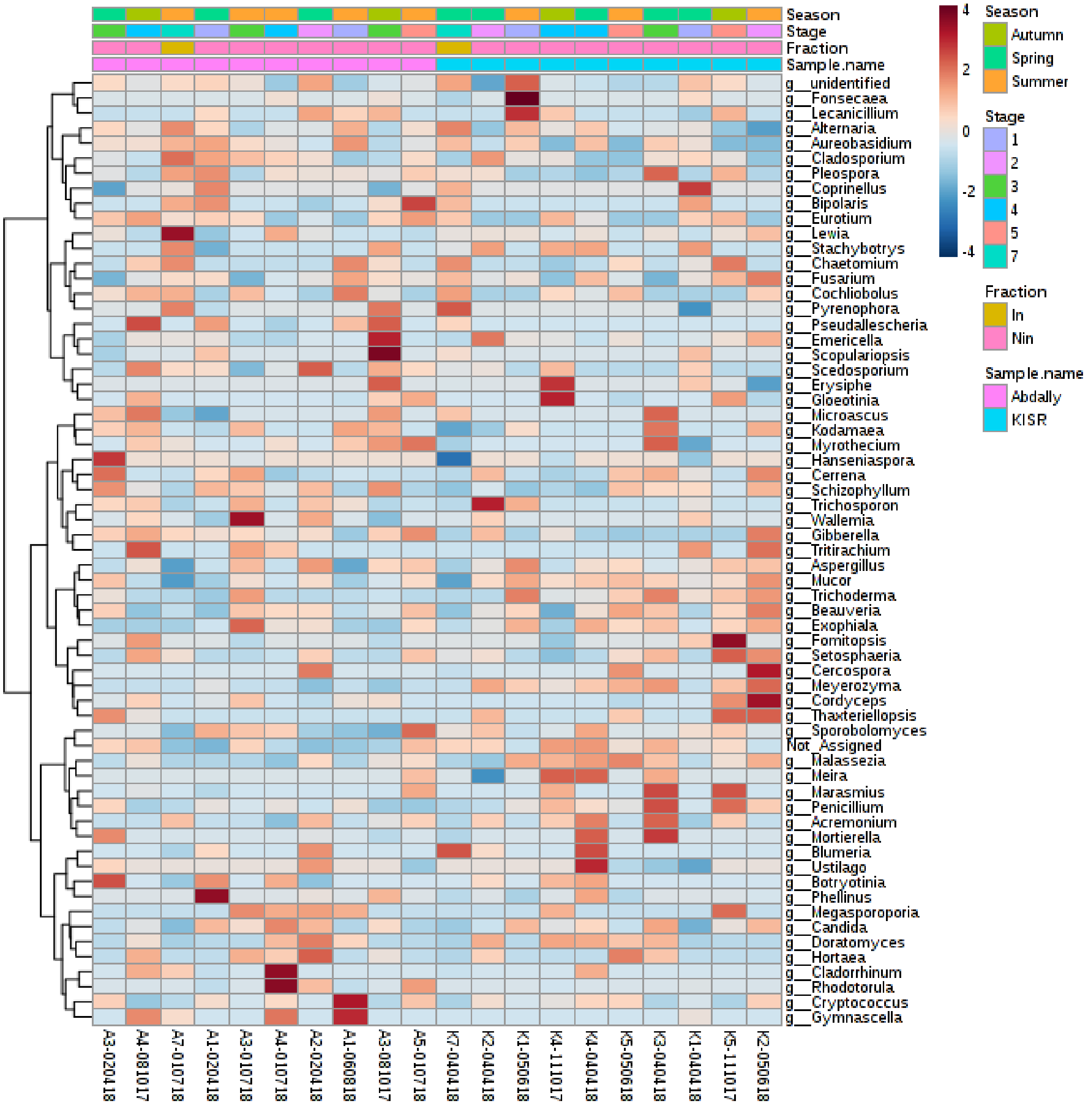

Figure S6b: Hierarchical clustering of fungal genera. Ward Algorithm was applied on the Euclidean distances to present the spatio-temporal variations with respect to the prevalence of all the detected fungal genera. Dark coloured boxes represent the high prevalence of a particular genus in a particular season at the specific site.
