# Supplementary material for: Spatio-temporal variations in bacterial and fungal community associated with dust aerosol in Kuwait
Source: PLoS One. 2020 Nov 5;15(11):e0241283. doi: 10.1371/journal.pone.0241283 (PMC7644028; doi:10.1371/journal.pone.0241283)
Supplement: S1 Table — Taxonomy and core microbiome analysis (S1a) Bacterial taxonomic classification at phylum level; (S1b) Bacterial taxonomic classification at class level; (S1c) Bacterial taxonomic classification at order level; (S1d) Bacterial taxonomic classification at family level; (S1e) Bacterial taxonomic classification at genus level; (S1f) Core bacteriome; (S1g) Fungal taxonomic classification at phylum level; (S1h) Fungal taxonomic classification at class level; (S1i) Fungal taxonomic classification at order level; (S1j) Fungal taxonomic classification at family level; (S1k) Fungal taxonomic classification at genus level; (S1l) Fungal taxonomic classification at species level; (S1m) Core fungal biome. (PDF) [file pone.0241283.s003.pdf]

Supplementary File S5: Taxonomy and Core Microbiome Analysis

Bacterial Taxonomy

Table S5a: Bacterial Taxonomic classification at Phylum level

	Taxa	Abundance	Percentage
1	p__Proteobacteria	212887	97.67
3	p__Actinobacteria	2455	1.13
2	p__Bacteroidetes	1816	0.83
4	p__Firmicutes	719	0.33
5	Unclassified	83	0.04

Table S5b: Bacterial Taxonomic Classification at Class level

	Taxa	Abundance	Percentage
1	c__Gammaproteobacteria	154928	71.04
3	c__Alphaproteobacteria	57835	26.52
4	c__Actinobacteria	2472	1.13
2	c__Bacteroidia	1832	0.84
6	c__Bacilli	606	0.28
8	Not_Assigned	294	0.13
5	c__Clostridia	86	0.04
7	c__Erysipelotrichia	27	0.01

Table S5c: Bacterial Taxonomic Classification at Order level

	Taxa	Abundance	Percentage
3	o__Betaproteobacteriales	94938	43.53
10	o__Pseudomonadales	51984	23.84
4	o__Caulobacterales	27903	12.79
12	o__Sphingomonadales	20246	9.28
11	o__Rhizobiales	8664	3.97
8	o__Oceanospirillales	2958	1.36
13	o__Xanthomonadales	2396	1.1
14	Others	1785	0.82
6	o__Cytophagales	1561	0.72
2	o__Alteromonadales	1414	0.65
7	o__Micrococcales	1360	0.62
1	Not_Assigned	1100	0.5
5	o__Corynebacteriales	929	0.43
9	o__Parvibaculales	842	0.39

Table S5d: Bacterial Taxonomic Classification at Family Level

	Taxa	Abundance	Percentage
1	f__Burkholderiaceae	94938	43.53
2	f__Pseudomonadaceae	48578	22.28
3	f__Caulobacteraceae	27903	12.79
4	f__Sphingomonadaceae	20246	9.28
5	f__Rhizobiaceae	6572	3.01
6	f__Moraxellaceae	3406	1.56
7	f__Halomonadaceae	2851	1.31
8	f__Xanthomonadaceae	2396	1.1
9	f__Hymenobacteraceae	1561	0.72
10	f__Shewanellaceae	1414	0.65
11	f__Devosiaceae	1286	0.59
12	Not_Assigned	1100	0.5
13	f__Corynebacteriaceae	929	0.43
14	f__Parvibaculaceae	842	0.39
15	f__Microbacteriaceae	690	0.32
16	f__Beijerinckiaceae	658	0.3
17	f__Micrococcaceae	484	0.22
18	f__Solimonadaceae	432	0.2
19	f__Staphylococcaceae	321	0.15
20	f__Bacillaceae	285	0.13
21	f__Flavobacteriaceae	271	0.12
22	f__Intrasporangiaceae	156	0.07
23	f__Propionibacteriaceae	153	0.07
24	f__Xanthobacteraceae	148	0.07
25	f__Rhodobacteraceae	137	0.06
26	f__Hahellaceae	82	0.04
27	f__Peptostreptococcaceae	49	0.02
28	f__Azospirillaceae	43	0.02
29	f__Clostridiaceae_1	37	0.02
30	f__Dermabacteraceae	30	0.01
31	f__Erysipelotrichaceae	27	0.01
32	f__SS1_B_06_26	25	0.01
33	f__Nocardioidaceae	15	0.01
34	f__Geodermatophilaceae	15	0.01

Table S5e: Bacterial Taxonomic Classification at Genus level

	Taxa	Abundance	Percentage
1	Not_Assigned	127725	58.57
2	g__Brevundimonas	27248	12.49
3	g__Sphingobium	7196	3.3
4	g__Sphingopyxis	6035	2.77
5	g__Pseudomonas	5448	2.5
6	g__Sphingomonas	5265	2.41
7	g__Massilia	5143	2.36
8	g__Acidovorax	4461	2.05
9	g__Allorhizobium	3840	1.76
10	g__Halomonas	2851	1.31
11	g__Mesorhizobium	2492	1.14
12	g__Novosphingobium	1750	0.8
13	g__Pseudoxanthomonas	1709	0.78
14	g__Enhydrobacter	1606	0.74
15	g__Pontibacter	1561	0.72
16	g__Shewanella	1414	0.65
17	g__Cavicella	1404	0.64
18	g__Devosia	1286	0.59
19	g__Achromobacter	1015	0.47
20	g__Corynebacterium_1	929	0.43
21	g__Parvibaculum	842	0.39
22	g__Delftia	822	0.38
23	g__Stenotrophomonas	687	0.32
24	g__Rhizobacter	585	0.27
25	g__Microvirga	484	0.22
26	g__uncultured	463	0.21
27	g__Variovorax	461	0.21
28	g__Azotobacter	447	0.2
29	g__Hydrocarboniphaga	432	0.2
30	g__Pseudarthrobacter	339	0.16
31	g__Staphylococcus	321	0.15
32	g__Bacillus	285	0.13
33	g__Salinimicrobium	271	0.12
34	g__Methylobacterium	174	0.08
35	g__Tetrasphaera	156	0.07
36	g__Cutibacterium	153	0.07
37	g__Bradyrhizobium	148	0.07
38	g__Kocuria	95	0.04
39	g__Hahella	82	0.04

40	g__Paracoccus	80	0.04
41	g__Ramlibacter	69	0.03
42	g__Micrococcus	50	0.02
43	g__Romboutsia	49	0.02
44	g__Skermanella	43	0.02
45	g__Noviherbaspirillum	40	0.02
46	g__Clostridium_sensu_stricto_1	37	0.02
47	g__Brachybacterium	30	0.01
48	g__Turicibacter	27	0.01
49	g__Aeromicrobium	15	0.01
50	g__Blastococcus	15	0.01

Table S5f: **Core Bacteriome:** The core Bacteriome analysis was performed on the set of 224 OTUs filtered through the MicrobiomeAnalyst. The below taxa with a sample prevalence of 20% and a relative abundance above 0.01 was obtained as the core bacteriome.

	**Prevelance**
g__Brevundimonas	1
g__Mesorhizobium	1
Not_Assigned	1
g__Allorhizobium	0.8
g__Massilia	0.8
g__Devosia	0.75
g__Pseudomonas	0.75
g__Achromobacter	0.65
g__Sphingopyxis	0.65
g__Azotobacter	0.45
g__Microvirga	0.4
g__Sphingomonas	0.4
g__Stenotrophomonas	0.35
g__Acidovorax	0.3
g__Cavicella	0.3
g__Novosphingobium	0.3
g__Parvibaculum	0.3
g__Pseudoxanthomonas	0.3
g__uncultured	0.3
g__Variovorax	0.3
g__Bacillus	0.25
g__Halomonas	0.25
g__Hydrocarboniphaga	0.25
g__Pseudarthrobacter	0.25

g__Sphingobium	0.25
g__Corynebacterium_1	0.2
g__Pontibacter	0.2
g__Rhizobacter	0.2
g__Shewanella	0.2
g__Bradyrhizobium	0.15
g__Enhydrobacter	0.15
g__Cutibacterium	0.1
g__Delftia	0.1
g__Hahella	0.05
g__Kocuria	0.05
g__Methylobacterium	0.05
g__Micrococcus	0.05
g__Noviherbaspirillum	0.05
g__Paracoccus	0.05
g__Ramlibacter	0.05
g__Salinimicrobium	0.05
g__Staphylococcus	0.05
g__Tetrasphaera	0.05
g__Aeromicrobium	0
g__Blastococcus	0
g__Brachybacterium	0
g__Clostridium_sensu_stricto_1	0
g__Romboutsia	0
g__Skermanella	0
g__Turicibacter	0

Fungal Taxonomy

Table S5g: Fungal Taxonomic classification at Phylum level

	Taxa	Abundance	Percentage
1	p__Ascomycota	347907	64.19
2	p__Basidiomycota	122714	22.64
3	Unclassified	64018	11.81
4	p__Zygomycota	7321	1.35

Table S5h: Fungal Taxonomic Classification at Class level

	Taxa	Abundance	Percentage
3	c__Dothideomycetes	190556	35.16
4	Not_Assigned	95622	17.64
8	c__Tremellomycetes	68808	12.7
5	c__Eurotiomycetes	52968	9.77
1	c__Sordariomycetes	36534	6.74
7	c__Agaricomycetes	33707	6.22
10	c__Saccharomycetes	25077	4.63
2	c__Incertae_sedis	24110	4.45
9	c__Leotiomycetes	8393	1.55
11	c__Microbotryomycetes	2775	0.51
6	c__Pezizomycetes	2166	0.4
14	c__unidentified	882	0.16
12	c__Wallemiomycetes	203	0.04
13	c__Ustilaginomycetes	83	0.02
15	c__Exobasidiomycetes	76	0.01

Table S5i: Fungal Taxonomic Classification at Order level

	Taxa	Abundance	Percentage
13	o__Capnodiales	104331	19.25
4	Not_Assigned	97407	17.97
3	o__Pleosporales	83150	15.34
9	o__Filobasidiales	66286	12.23
5	o__Eurotiales	48019	8.86
12	o__Hypocreales	29173	5.38
11	o__Saccharomycetales	25085	4.63
7	o__Polyporales	23711	4.38
2	o__Malasseziales	16170	2.98
20	o__Agaricales	8894	1.64

10	o__Helotiales	7531	1.39
24	o__Mucorales	3979	0.73
1	o__Microascales	3817	0.7
17	o__Chaetothyriales	3380	0.62
22	o__Mortierellales	3329	0.61
27	o__Dothideales	3159	0.58
15	o__Sporidiobolales	2784	0.51
16	o__Sordariales	2536	0.47
23	o__Tremellales	2116	0.39
6	o__Pezizales	1918	0.35
19	o__Onygenales	1244	0.23
26	o__unidentified	1147	0.21
21	o__Erysiphales	911	0.17
14	o__Hymenochaetales	818	0.15
25	o__Incertae_sedis	699	0.13
18	o__Wallemiales	204	0.04
28	o__Ustilaginales	87	0.02
8	o__Xylariales	75	0.01

Table S5j: Fungal Taxonomic Classification at Family level

	Taxa	Abundance	Percentage
2	Not_Assigned	149342	27.56
8	f__Mycosphaerellaceae	97185	17.93
6	f__Filobasidiaceae	66239	12.22
3	f__Pleosporaceae	62007	11.44
4	f__Trichocomaceae	48028	8.86
7	f__Incertae_sedis	36588	6.75
10	f__unidentified	18877	3.48
21	f__Nectriaceae	8997	1.66
35	f__Schizophyllaceae	8622	1.59
19	f__Sporormiaceae	5822	1.07
27	f__Mucoraceae	3993	0.74
1	f__Microascaceae	3766	0.69
12	f__Herpotrichiellaceae	3386	0.62
24	f__Mortierellaceae	3333	0.61
31	f__Dothioraceae	2903	0.54
15	f__Clavicipitaceae	2395	0.44
38	f__Teratosphaeriaceae	2286	0.42
25	f__Trichosporonaceae	2120	0.39
37	f__Hypocreaceae	2000	0.37

28	f__Polyporaceae	1915	0.35
11	f__Chaetomiaceae	1716	0.32
5	f__Pezizaceae	1530	0.28
33	f__Tubeufiaceae	1268	0.23
16	f__Onygenaceae	1087	0.2
18	f__Fomitopsidaceae	1062	0.2
23	f__Erysiphaceae	908	0.17
9	f__Hymenochaetaceae	828	0.15
13	f__Cordycipitaceae	777	0.14
26	f__Lasiosphaeriaceae	734	0.14
22	f__Debaryomycetaceae	707	0.13
29	f__Sclerotiniaceae	591	0.11
34	f__Ascobolaceae	212	0.04
14	f__Wallemiaceae	210	0.04
17	f__Gymnoascaceae	209	0.04
20	f__Marasmiaceae	130	0.02
32	f__Ustilaginaceae	87	0.02
36	f__Saccharomycodaceae	54	0.01
30	f__Psathyrellaceae	46	0.01

Table S5k: Fungal Taxonomic Classification at Genus level

	Taxa	Abundance	Percentage
2	Not_Assigned	172419	31.81
5	g__unidentified	102488	18.91
9	g__Cryptococcus	65663	12.12
3	g__Alternaria	46554	8.59
7	g__Aspergillus	36298	6.7
11	g__Candida	16577	3.06
12	g__Cladosporium	15920	2.94
56	g__Schizophyllum	8622	1.59
31	g__Fusarium	7511	1.39
10	g__Gloeotinia	6920	1.28
4	g__Penicillium	6216	1.15
18	g__Pleospora	4189	0.77
42	g__Mucor	3993	0.74
37	g__Mortierella	3333	0.61
30	g__Eurotium	3010	0.56
52	g__Aureobasidium	2903	0.54
8	g__Cochliobolus	2661	0.49
23	g__Malassezia	2609	0.48

24	g__Rhodotorula	2299	0.42
63	g__Hortaea	2286	0.42
40	g__Trichosporon	2120	0.39
46	g__Exophiala	1944	0.36
38	g__Beauveria	1609	0.3
55	g__Gibberella	1486	0.27
45	g__Cerrena	1435	0.26
39	g__Scedosporium	1420	0.26
49	g__Chaetomium	1369	0.25
54	g__Thaxteriellopsis	1268	0.23
28	g__Setosphaeria	1191	0.22
61	g__Cercospora	1077	0.2
27	g__Fomitopsis	1062	0.2
60	g__Trichoderma	999	0.18
13	g__Phellinus	828	0.15
20	g__Lecanicillium	777	0.14
6	g__Emericella	739	0.14
35	g__Blumeria	731	0.13
34	g__Meyerozyma	707	0.13
41	g__Doratomyces	668	0.12
22	g__Myrothecium	643	0.12
44	g__Tritirachium	602	0.11
47	g__Botryotinia	591	0.11
33	g__Cordyceps	579	0.11
19	g__Kodamaea	570	0.11
51	g__Acremonium	560	0.1
62	g__Megasporoporia	480	0.09
14	g__Sporobolomyces	453	0.08
17	g__Fonsecaea	438	0.08
48	g__Cladorrhinum	363	0.07
1	g__Microascus	302	0.06
36	g__Bipolaris	290	0.05
15	g__Pseudallescheria	276	0.05
32	g__Lewia	263	0.05
16	g__Stachybotrys	213	0.04
21	g__Wallemia	210	0.04
26	g__Gymnascella	209	0.04
58	g__Meira	183	0.03
64	g__Erysiphe	177	0.03
43	g__Pyrenophora	161	0.03
25	g__Metarhizium	130	0.02

29	g__Marasmius	130	0.02
53	g__Ustilago	87	0.02
57	g__Hanseniaspora	54	0.01
59	g__Scopulariopsis	49	0.01
50	g__Coprinellus	46	0.01

Table S5l: Fungal Taxonomic classification at species level

	Taxa	Abundance	Percentage
1	Not_Assigned	225762	41.66
2	s__unculturedCladosporium	80188	14.8
3	s__Alternaria_brassicae	46447	8.57
4	s__Cryptococcus_oeirensis	30610	5.65
5	s__Cryptococcus_albidus	21755	4.01
6	s__Candida_parapsilosis	13002	2.4
7	s__Aspergillus_gracilis	10667	1.97
8	s__Aspergillus_penicillioides	9536	1.76
9	s__unculturedfungus	9376	1.73
10	s__Schizophyllum_commune	8622	1.59
11	s__Gloeotinia_temulenta	6920	1.28
12	s__Cryptococcus_carnescens	5205	0.96
13	s__Capnodiales_sp_TR006	4385	0.81
14	s__Aspergillus_versicolor	3060	0.56
15	s__Cryptococcus_stepposus	2738	0.51
16	s__Aureobasidium_pullulans	2702	0.5
17	s__unculturedMalassezia	2584	0.48
18	s__Cryptococcus_laurentii	2390	0.44
19	s__Malassezia_globosa	2334	0.43
20	s__Rhodotorula_mucilaginosa	2299	0.42
21	s__Hortaea_werneckii	2286	0.42
22	s__Trichosporon_asahii	2120	0.39
23	s__Cryptococcus_magnus	2083	0.38
24	s__Exophiala_dermatitidis	1944	0.36
25	s__Pleospora_herbarum	1846	0.34
26	s__Candida_sp_F15	1812	0.33
27	s__unculturedEurotium	1765	0.33
28	s__Aspergillus_niger	1732	0.32
29	s__Candida_tropicalis	1639	0.3
30	s__Beauveria_bassiana	1609	0.3
31	s__Fusarium_oxysporum	1594	0.29
33	s__Eurotium_niveoglaucum	1542	0.28

32	s__Pezizaceae_sp_F13	1530	0.28
35	s__Cerrena_unicolor	1435	0.26
34	s__Scedosporium_prolificans	1420	0.26
36	s__Chaetomium_globosum	1369	0.25
37	s__Aspergillus_peyronelii	1270	0.23
38	s__Thaxteriellopsis_lignicola	1268	0.23
39	s__Cochliobolus_sp_FSU9312	1210	0.22
40	s__Setosphaeria_rostrata	1191	0.22
42	s__Cercospora_capsici	1077	0.2
41	s__Fomitopsis_sp_CLF_T	1062	0.2
43	s__Gibberella_intermedia	977	0.18
44	s__Cochliobolus_sp_007 L 1_1	896	0.17
45	s__Phellinus_noxius	828	0.15
47	s__Cryptococcus_dimennae	777	0.14
48	s__Lecanicillium_fusisporum	777	0.14
46	s__Emericella_nidulans	739	0.14
49	s__Blumeria_graminis	731	0.13
53	s__Tritirachium_sp_IAM_14522	602	0.11
52	s__Cladosporium_sp_B09	591	0.11
54	s__Botryotinia_fuckeliana	591	0.11
51	s__Penicillium_capsulatum	573	0.11
50	s__Kodamaea_ohmeri	570	0.11
56	s__Cochliobolus_sativus	555	0.1
55	s__Cordyceps_chlamydosporia	554	0.1
57	s__Gibberella_zeae	509	0.09
58	s__Megasporoporia_sp_HJL_2012d	480	0.09
59	s__Fonsecaea_pedrosoi	438	0.08
60	s__Myrothecium_verrucaria	435	0.08
61	s__Cladorrhinum_bulbillosum	363	0.07
64	s__Cladosporium_sp_ZJ_2008016	342	0.06
63	s__Doratomyces_sp_HZ_10	322	0.06
62	s__Microascus_sp_4L1	302	0.06
65	s__Pseudallescheria_fimeti	276	0.05
67	s__Malassezia_sympodialis	275	0.05
66	s__Pezizomycetes_sp_BLD9	252	0.05
68	s__Stachybotrys_chlorohalonata	213	0.04
70	s__Gymnascella_aurantiaca	209	0.04
69	s__Myrothecium_gramineum	208	0.04
72	s__Aspergillus_terreus	185	0.03
75	s__Meira_sp_VegaE2_32	183	0.03
76	s__Erysiphe_convolvuli	177	0.03

71	s__Penicillium_chrysogenum	166	0.03
74	s__Pyrenophora_graminea	161	0.03
73	s__Pezizales_sp_P10	149	0.03
79	s__Metarhizium_anisopliae	130	0.02
80	s__Marasmius_leveilleanus	130	0.02
78	s__unculturedThielavia	128	0.02
83	s__Sporobolomyces_roseus	126	0.02
82	s__Candida_orthopsilosis	124	0.02
77	s__Cryptococcus_sp_HB_1222	105	0.02
81	s__Ustilago_hordei	87	0.02
86	s__Acremonium_implicatum	80	0.01
84	s__Hypocreales_sp_SCSGAF0072	77	0.01
88	s__Cladosporium_sp_F128	62	0.01
87	s__Hanseniaspora_uvarum	54	0.01
85	s__Coprinellus_sp_Daams_72_68a	46	0.01
89	s__Aspergillus_caespitosus	19	0

Table S5m **Core Fungal Biome:** The core fungal biome analysis was performed on the set of 371 OTUs filtered through the MicrobiomeAnalyst. A set of 65 taxa with a sample prevalence of 20% and a relative abundance above 0.01 was obtained as the core microbiome.

	Prevelance	
Not_Assigned	1	100
g__unidentified	1	100
g__Cryptococcus	1	100
g__Alternaria	1	100
g__Aspergillus	0.9	90
g__Cladosporium	0.75	75
g__Fusarium	0.6	60
g__Candida	0.55	55
g__Schizophyllum	0.3	30
g__Penicillium	0.25	25
g__Pleospora	0.2	20
g__Mucor	0.2	20
g__Cochliobolus	0.2	20
g__Aureobasidium	0.2	20
g__Scedosporium	0.15	15
g__Mortierella	0.15	15
g__Malassezia	0.15	15
g__Hortaea	0.15	15
g__Thaxteriellopsis	0.1	10

g__Gloeotinia	0.1	10
g__Eurotium	0.1	10
g__Chaetomium	0.1	10
g__Cerrena	0.1	10
g__Tritirachium	0.05	5
g__Trichosporon	0.05	5
g__Setosphaeria	0.05	5
g__Rhodotorula	0.05	5
g__Phellinus	0.05	5
g__Lecanicillium	0.05	5
g__Gibberella	0.05	5
g__Fonsecaea	0.05	5
g__Fomitopsis	0.05	5
g__Exophiala	0.05	5
g__Emericella	0.05	5
g__Cordyceps	0.05	5
g__Cercospora	0.05	5
g__Blumeria	0.05	5
g__Beauveria	0.05	5
g__Wallemia	0	0
g__Ustilago	0	0
g__Trichoderma	0	0
g__Stachybotrys	0	0
g__Sporobolomyces	0	0
g__Scopulariopsis	0	0
g__Pyrenophora	0	0
g__Pseudallescheria	0	0
g__Myrothecium	0	0
g__Microascus	0	0
g__Meyerozyma	0	0
g__Meira	0	0
g__Megasporoporia	0	0
g__Marasmius	0	0
g__Lewia	0	0
g__Kodamaea	0	0
g__Hanseniaspora	0	0
g__Gymnascella	0	0
g__Erysiphe	0	0
g__Doratomyces	0	0
g__Coprinellus	0	0
g__Cladorrhinum	0	0

g__Botryotinia	0	0
g__Bipolaris	0	0
g__Acremonium	0	0
