# Supplementary material for: Spatio-temporal variations in bacterial and fungal community associated with dust aerosol in Kuwait
Source: PLoS One. 2020 Nov 5;15(11):e0241283. doi: 10.1371/journal.pone.0241283 (PMC7644028; doi:10.1371/journal.pone.0241283)
Supplement: S2 Table — Linear discriminant analysis (S2a) LDA analysis of bacterial genus according to the site; (S2b); LDA analysis of bacterial genus according to the season; (S2c) LDA analysis of bacterial genus according to the stage; (S2d) LDA analysis of fungal genus according to the site; (S2e) LDA analysis of fungal genus according to the season; (S2f) LDA analysis of fungal genus according to the stage. (PDF) [file pone.0241283.s004.pdf]

Linear Discriminant Analysis

Table S7a: LDA analysis of bacterial genus according to the site

The table below shows at most 500 features ranked by their p values, with significant features highlighted in orange.

Name	Pvalues	FDR	Remote	Urban	LDAscore	View
g__Pontibacter	5.4399E-4	0.0272	154040.0	2404.2	4.88	Details
g__Romboutsia	0.0015463	0.038657	4155.0	156.51	3.3	Details
g__Clostridium_sensu_stricto_1	0.0083345	0.13891	3184.5	161.63	3.18	Details
g__Turicibacter	0.012891	0.14921	3107.1	96.699	3.18	Details
Not_Assigned	0.016703	0.14921	4050600.0	7360400.0	-6.22	Details
g__Acidovorax	0.017905	0.14921	360830.0	75175.0	5.15	Details
g__Noviherbaspirillum	0.027538	0.17808	4690.8	181.14	3.35	Details
g__Rhizobacter	0.028493	0.17808	49717.0	6918.3	4.33	Details
g__Salinimicrobium	0.033952	0.18089	28357.0	205.57	4.15	Details
g__Ramlibacter	0.036178	0.18089	5849.7	245.49	3.45	Details
g__Brevundimonas	0.044083	0.20038	1990000.0	629540.0	5.83	Details
g__Parvibaculum	0.074091	0.27571	77982.0	8454.5	4.54	Details
g__Cavicella	0.082151	0.27571	126880.0	14884.0	4.75	Details
g__Azotobacter	0.087375	0.27571	14762.0	23842.0	-3.66	Details

Xia Lab @ McGill (last updated 2020-01-08)

Table S7b: LDA analysis of bacterial genus according to the season

The table below shows at most 500 features ranked by their p values, with significant features highlighted in orange.

Name	Pvalues	FDR	Spring	Summer	Winter	LDAscore	View
g__Novosphingobium	3.4787E-4	0.0044121	0.0	10061.0	286740.0	5.17	Details
g__Gaviella	4.8376E-4	0.0044121	0.0	4562.2	258830.0	5.1	Details
g__Sphingomonas	5.2894E-4	0.0044121	601.93	53246.0	884660.0	5.65	Details
g__Micrococcus	5.6894E-4	0.0044121	0.0	5929.9	279.88	3.47	Details
g__uncultured	7.4348E-4	0.0044121	0.0	4188.9	73878.0	4.87	Details
g__Staphylococcus	9.9809E-4	0.0044121	142.8	37877.0	178.89	4.27	Details
g__Massilia	0.0010393	0.0044121	121980.0	472700.0	9888.8	5.37	Details
g__Sphingobium	0.0010399	0.0044121	65.393	1758.2	1322900.0	5.82	Details
g__Aeromicrobium	0.0010401	0.0044121	2007.9	273.59	0.0	3.0	Details
g__Halomonas	0.0010404	0.0044121	2484.3	322550.0	1961.2	5.2	Details
g__Sphingopyxis	0.001138	0.0044121	538.83	562740.0	210500.0	5.45	Details
g__Enhydrobacter	0.0012029	0.0044121	162.32	179120.0	567.21	4.95	Details
g__Achromobacter	0.0012049	0.0044121	160.84	60617.0	69746.0	4.65	Details
g__Shewanella	0.0013604	0.0044121	992.95	158400.0	829.16	4.89	Details
g__Hydrocarboniphaga	0.0013823	0.0044121	130.79	668.7	81022.0	4.61	Details
g__Pseudoxanthomonas	0.0014119	0.0044121	40.59	8806.1	294880.0	5.17	Details
g__Rhizobacter	0.0015566	0.0046078	181.09	3249.1	99829.0	4.7	Details
g__Terrisphaera	0.0021324	0.0059284	256.64	16586.0	358.17	3.91	Details
g__Cutibacterium	0.0023132	0.0060876	0.0	18807.0	366.48	3.96	Details
g__Pseudarthrobacter	0.0024582	0.0061464	26661.0	13592.0	627.04	4.11	Details
g__Bradyrhizobium	0.0031594	0.0075223	268.51	6738.3	14024.0	3.84	Details
g__Morovinga	0.0042144	0.0095781	33969.0	24160.0	266.07	4.21	Details
g__Acidovorax	0.0049058	0.010467	473.96	47217.0	738870.0	5.57	Details
g__Paracoccus	0.0050241	0.010467	2419.0	6635.1	0.0	3.52	Details
g__Parvibaculum	0.0052754	0.010561	3472.0	21360.0	103900.0	4.7	Details
g__Variovorax	0.0066984	0.012604	124.0	14423.0	57724.0	4.46	Details
g__Allorhizobium_Neorhizob	0.0081062	0.01518	224090.0	226900.0	18287.0	5.01	Details
g__Methylobacterium	0.010151	0.018181	427.81	21900.0	188.22	4.04	Details
g__Skermanella	0.019519	0.024171	1033.1	3116.4	0.0	3.19	Details
g__Hahella	0.022227	0.037046	9458.5	3932.7	0.0	3.67	Details
Not_Assigned	0.02328	0.037549	7746100.0	6346000.0	4064500.0	6.26	Details
g__Delftia	0.030825	0.047896	85.398	3029.3	154650.0	4.89	Details
g__Pseudomonas	0.033332	0.050504	52830.0	309550.0	442940.0	5.28	Details
g__Rombouisia	0.03815	0.056103	1591.7	147.11	5219.5	3.4	Details
g__Brachybacterium	0.046014	0.065734	550.79	3479.3	175.89	3.22	Details
g__Kocuria	0.056427	0.07937	1030.7	6976.8	288.07	3.52	Details
g__Blastococcus	0.061865	0.083723	560.11	1150.8	175.58	2.7	Details
g__Salinimicrobium	0.073859	0.097314	35844.0	620.76	0.0	4.25	Details
g__Novihalobaspirillum	0.08409	0.10781	4480.9	1605.4	0.0	3.35	Details
g__Devosia	0.091373	0.11422	24635.0	71940.0	85040.0	4.48	Details

Table 7c: LDA analysis of bacterial genus according to the stage

The table below shows at most 500 features ranked by their p values, with significant features highlighted in orange.

Name	Pvalues	FDR	1	2	3	4	5	LDAscore	View
denovo20023	0.098872	0.99831	7191.9	0.0	4009.4	0.0	3128.3	3.56	Details
denovo2056	0.14558	0.99831	2008.3	5629.4	966.11	2210.6	2906.4	3.37	Details
denovo28732	0.1664	0.99831	2949.0	2924.0	502.74	2348.2	302.21	3.11	Details
denovo27389	0.18013	0.99831	10167.0	40749.0	5915.9	12893.0	3337.9	4.27	Details
denovo7813	0.18864	0.99831	539.75	1385.1	1402.6	38287.0	2342.8	4.28	Details
denovo18480	0.19329	0.99831	0.0	67507.0	19320.0	5256.8	38108.0	4.46	Details
denovo22017	0.21466	0.99831	0.0	7264.2	0.0	6951.4	11101.0	2.74	Details
denovo18847	0.21969	0.99831	525.92	349.55	2187.5	858.34	804.31	2.96	Details
denovo13631	0.22771	0.99831	6000.0	893.78	75.844	996.62	2160.0	3.39	Details
denovo24124	0.2291	0.99831	2642.3	4626.4	13490.0	84842.0	5965.4	4.49	Details
denovo123	0.25136	0.99831	1779.6	305.17	335.21	732.46	692.44	2.87	Details
denovo9611	0.25476	0.99831	36845.0	1756.8	4368.4	0.0	637.96	4.27	Details
denovo4658	0.25946	0.99831	1436.0	1701.1	1296.5	2296.4	368.84	2.96	Details
denovo3896	0.27502	0.99831	4826.2	999.44	15250.0	4501.9	15942.0	3.87	Details
denovo27114	0.28113	0.99831	4349.3	7886.0	4761.9	6233.8	23137.0	3.97	Details
denovo31045	0.28676	0.99831	6145.7	5667.2	2786.7	4551.9	1395.6	3.36	Details
denovo9207	0.29528	0.99831	1342.6	1836.8	1264.7	699.89	527.33	2.82	Details
denovo23495	0.29549	0.99831	1667.3	684.72	4362.6	1475.0	1031.4	3.27	Details
denovo8574	0.30623	0.99831	640.01	116.52	1517.7	1229.0	464.54	2.65	Details
denovo21341	0.30855	0.99831	16515.0	466.07	31809.0	35957.0	47021.0	4.37	Details

Table 7d: LDA analysis of fungal genus according to the site

The table below shows at most 500 features ranked by their p values, with significant features highlighted in orange.

Name	Pvalues	FDR	Remote	Urban	LDAscore	View
g__Meyerozyma	0.0033641	0.21194	1355.0	25517.0	4.08	Details
g__Trichoderma	0.014795	0.41881	7476.5	29273.0	4.04	Details
g__Gibberella	0.022311	0.41881	32626.0	21468.0	-3.75	Details
g__Pseudallescheria	0.026591	0.41881	9864.7	1027.3	-3.65	Details
g__Alternaria	0.04125	0.4443	1096000.0	625170.0	-5.37	Details
g__Lewia	0.044348	0.4443	6722.0	2222.7	-3.35	Details
g__Penicillium	0.049366	0.4443	34232.0	193010.0	4.9	Details
g__Malassezia	0.069223	0.4875	31936.0	63611.0	4.2	Details
g__Mucor	0.069642	0.4875	46024.0	101250.0	4.44	Details
g__Scedosporium	0.084767	0.53403	41126.0	9310.6	-4.2	Details
g__Meira	0.11193	0.57755	419.62	2878.4	3.09	Details
g__Gymnascella	0.11193	0.57755	7349.1	688.23	-3.52	Details
g__Myrothecium	0.13512	0.57755	15709.0	7348.6	-3.62	Details
g__Exophiala	0.13897	0.57755	35724.0	33986.0	-2.94	Details
g__unidentified	0.15093	0.57755	2419600.0	1358800.0	-5.72	Details
g__Wallemia	0.15585	0.57755	6005.1	1382.1	-3.36	Details
g__Rhodotorula	0.15585	0.57755	83378.0	742.44	-4.62	Details
g__Erysiphe	0.19652	0.65906	2817.7	3304.2	2.39	Details
Not_Assigned	0.19876	0.65906	2713200.0	3651200.0	5.67	Details

Xia Lab @ McGill (last updated 2020-01-08)

Table S7e: LDA analysis of fungal genus according to the season

The table below shows at most 500 features ranked by their p values, with significant features highlighted in orange.

Name	Pvalues	FDR	Spring	Summer	Winter	LDAscore	View
g__Gloeotinia	0.001995	0.12568	39.89	11788.0	493900.0	5.39	Details
g__Fomitopsis	0.019938	0.30911	1448.9	204.75	77758.0	4.59	Details
g__Mortierella	0.021994	0.30911	153930.0	0.0	72.249	4.89	Details
g__Blumeria	0.023584	0.30911	35182.0	162.54	0.0	4.25	Details
g__Chaetomium	0.024533	0.30911	17383.0	12099.0	58882.0	4.37	Details
g__Beauveria	0.04735	0.49717	22123.0	51171.0	11788.0	4.29	Details
g__Ustilago	0.064598	0.55144	3489.9	310.45	0.0	3.24	Details
g__Exophiala	0.070025	0.55144	23410.0	66996.0	8169.4	4.47	Details
g__Scopulariopsis	0.11401	0.72661	1077.8	0.0	934.57	2.73	Details
g__Eurotium	0.12305	0.72661	39733.0	31402.0	113250.0	4.61	Details
g__Lecanicillium	0.12756	0.72661	7863.1	17833.0	18480.0	3.73	Details
g__Stachybotrys	0.1478	0.72661	5245.2	914.31	4983.8	3.34	Details
g__Cochliobolus	0.16421	0.72661	28852.0	43930.0	94745.0	4.52	Details
g__Aureobasidium	0.17237	0.72661	76667.0	35998.0	46010.0	4.31	Details
g__Botryotinia	0.173	0.72661	20892.0	3595.7	5249.8	3.94	Details
g__Coprinellus	0.18701	0.73636	2620.1	40.636	505.74	3.11	Details
g__Cladosporium	0.21813	0.76219	285040.0	186710.0	452920.0	5.12	Details
g__Pseudallescheria	0.23574	0.76219	2958.7	1116.0	15488.0	3.86	Details
g__Cryptococcus	0.24545	0.76219	1218600.0	1254200.0	1161800.0	4.66	Details
[illegible]	[illegible]	[illegible]	[illegible]	[illegible]	[illegible]	[illegible]	[illegible]

Table S7f: LDA analysis of fungal genus according to the stage

The table below shows at most 500 features ranked by their p values, with significant features highlighted in orange.

Name	Pvalues	FDR	1	2	3	4	5	7	View
g__Cladorrhinum	0.02582	0.55038	837.32	0.0	0.0	32442.0	0.0	4.21	Details
g__Aureobasidium	0.041962	0.55038	99423.0	15672.0	55025.0	56744.0	9946.5	4.65	Details
g__Cercospora	0.045577	0.55038	0.0	116530.0	0.0	0.0	11143.0	4.77	Details
g__Beauveria	0.060552	0.55038	11627.0	65875.0	34869.0	16015.0	46920.0	4.5	Details
g__Candida	0.088018	0.55038	163850.0	479090.0	175530.0	788550.0	38081.0	5.59	Details
g__Setosphaeria	0.088309	0.55038	1098.2	38735.0	6892.5	22841.0	56750.0	4.44	Details
g__Pyrenophora	0.092833	0.55038	3029.9	123.01	4289.8	0.0	0.0	3.92	Details
g__Cerrena	0.096673	0.55038	9922.2	48848.0	80243.0	2303.2	7400.0	4.6	Details
g__Coprinellus	0.0983	0.55038	3833.1	0.0	723.92	0.0	0.0	3.28	Details
g__Kodamaea	0.098683	0.55038	9402.9	6173.4	31416.0	9047.6	0.0	4.2	Details
g__Schizophyllum	0.1009	0.55038	94516.0	182010.0	545010.0	17525.0	33719.0	5.44	Details
g__Doratomyces	0.10483	0.55038	4067.7	32018.0	5251.4	25079.0	3786.0	4.19	Details
g__Alternaria	0.12504	0.56168	1256500.0	352250.0	769490.0	892410.0	515460.0	5.75	Details
g__Microascus	0.13512	0.56168	837.32	0.0	14938.0	9875.9	0.0	3.87	Details
g__Fusarium	0.1444	0.56168	231630.0	171260.0	51884.0	78716.0	92084.0	4.98	Details
g__Scopulariopsis	0.15391	0.56168	1217.7	0.0	1542.4	0.0	0.0	2.89	Details
g__Lewia	0.15716	0.56168	1691.3	5476.4	2682.4	4097.2	509.27	3.96	Details
g__Blumeria	0.16048	0.56168	4856.4	27241.0	0.0	25471.0	0.0	4.3	Details
g__unidentified	0.1836	0.60877	2273700.0	940860.0	1616900.0	1704500.0	1206100.0	6.25	Details
g__Chaetomium	0.19692	0.62028	22436.0	0.0	8263.5	11249.0	50037.0	4.7	Details
