# Supplementary material for: Spatio-temporal variations in bacterial and fungal community associated with dust aerosol in Kuwait
Source: PLoS One. 2020 Nov 5;15(11):e0241283. doi: 10.1371/journal.pone.0241283 (PMC7644028; doi:10.1371/journal.pone.0241283)
Supplement: S3 Table — Seasonal diversity in relative abundances (a) Seasonal diversity of predominant bacterial genera; (b) Seasonal diversity of predominant fungal genera. (PDF) [file pone.0241283.s005.pdf]

Table S8a: Seasonal Diversity of predominant bacterial genera

Genus	Autumn;Remote	Autumn;Urban	Spring;Remote	Spring;Urban	Summer;Remote	Summer;Urban
g__Acidovorax	0.09525	**0.04074**	0.00015	0.00002	0.01352	0.00000
g__Allorhizobium	0.00055	0.00083	0.03153	0.01672	0.03114	0.01784
g__Brevundimonas	0.07454	0.01014	**0.24163**	**0.04969**	**0.28571**	**0.09455**
g__Halomonas	0.00028	0.00009	0.00028	0.00011	0.00162	0.05080
g__Massilia	0.00098	0.00000	0.01395	0.01211	0.05148	0.04559
g__Mesorhizobium	0.00896	0.01014	0.00893	0.00863	0.00505	0.02094
g__Pseudomonas	0.06255	0.01294	0.00315	0.00867	0.07157	0.00617
g__Sphingobium	**0.19738**	0.03239	0.00000	0.00002	0.00061	0.00000
g__Sphingomonas	0.14458	0.00408	0.00003	0.00014	0.00242	0.00664
g__Sphingopyxis	0.02545	0.01748	0.00003	0.00005	0.04848	0.05915
Not_Assigned	0.15795	0.78244	0.61219	0.89675	0.43895	0.58624
Others	0.23154	0.08873	0.08812	0.00709	0.04946	0.11209

Figures highlighted in bold depict the dominant forms

Table S8b: Seasonal Diversity of predominant fungal genera

Genus	Autumn;Remote	Autumn;Urban	Spring;Remote	Spring;Urban	Summer;Remote	Summer;Urban
g__Alternaria	0.15622	0.05029	**0.10175**	0.08337	**0.08113**	0.03566
g__Aspergillus	0.05768	0.05727	0.07798	0.05365	0.03544	0.13380
g__Candida	0.01033	0.01470	0.05470	0.01431	0.06727	0.01557
g__Cladosporium	0.03578	0.05760	0.02047	0.03389	0.02563	0.01041
g__Cryptococcus	**0.17672**	0.02641	0.06106	**0.15711**	0.07168	**0.19382**
g__Fusarium	0.02750	0.00663	0.00880	0.01344	0.00886	0.01562
g__Gloeotinia	0.00608	**0.11430**	0.00000	0.00000	0.00200	0.00002
g__Penicillium	0.00219	0.04950	0.00652	0.00861	0.00181	0.01700
g__Schizophyllum	0.04146	0.00066	0.04696	0.00292	0.00438	0.00922
g__unidentified	0.10383	0.19513	0.27920	0.13105	0.31550	0.10965
Not_Assigned	0.27258	0.33681	0.23071	0.38583	0.30320	0.35071
Others	0.10964	0.09070	0.11185	0.11583	0.08309	0.10851

Figures highlighted in bold depict the dominant forms
