# Supplementary material for: Spatio-temporal variations in bacterial and fungal community associated with dust aerosol in Kuwait
Source: PLoS One. 2020 Nov 5;15(11):e0241283. doi: 10.1371/journal.pone.0241283 (PMC7644028; doi:10.1371/journal.pone.0241283)
Supplement: S1 Appendix — S1a Table. Basic sequencing statistics and QIIME cut-off; S1b Table: Default parameters set in MicroBiomeAnalyst; S1a Fig: Library size overview of (i) Bacterial (ii) Fungal sequences; S1b Fig: Data rarefaction curves of (i) Bacterial and (ii) Fungal sequences. (PDF) [file pone.0241283.s007.pdf]

Supplementary File S4: Raw sequence quality and filtering parameters for OTU picking

Table S4a. Basic Sequencing Statistics and QIIME cut-off

Description	Bacterial	Fungal
Total Paired end Reads	38343-87060bp	82271-155807bp
GC Content	52.41-54.46%	49.65-55.91%
Phred Score	29.63-34-20	30.91-35.79
Phred Quality Distribution>= Q30	63.98-84.82%	69.85-90.54
Total Consensus Sequences	27834-65124	33904-84882
Consensus Sequences after Chimera Removal	14470-53121	32620-83666
Total Pre-processed Consensus	558721	1080482
Total OTUs Picked	31269	9312
Total Filtered OTUs(<5 reads)	29147	7132
Total Filtered OTUs(after filtering <5 reads)	2122	2180

Table S4b: Default parameters set in MicroBiomeAnalyst

Description	Bacterial	Fungal
OTUs in Input file	2115	2180
OTUs with ≥ 2 counts	1874	1334
Total read counts	517727	1033343
Average counts per sample	25886	51667
Maximum counts per sample	51453	80267
Minimum counts per sample	11766	30277
Low Count Filter	4	4
Prevalence	20%	20%
Inter quartile range	10%`	10%
Total OTUs retained	224	371

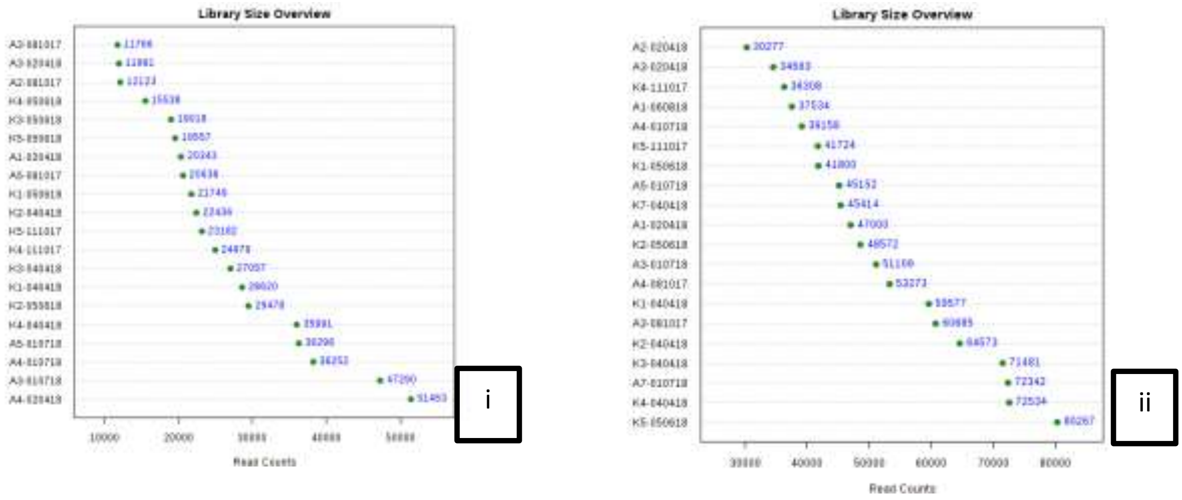

Figure S4a: Library Size Overview of (i) Bacterial (ii) Fungal Sequences

Data Rarefaction:

We performed the rarefaction to bring all the samples at a single sequence level. For bacterial samples the sequence size was normalized at 10898 and for fungal samples it was rarified at 27098. For the former we obtained a plateau beginning at 5000 whereas the plateau began at 10000 in case of fungal sequences. It was observed that beyond these levels there was little or negligible increase in species richness.

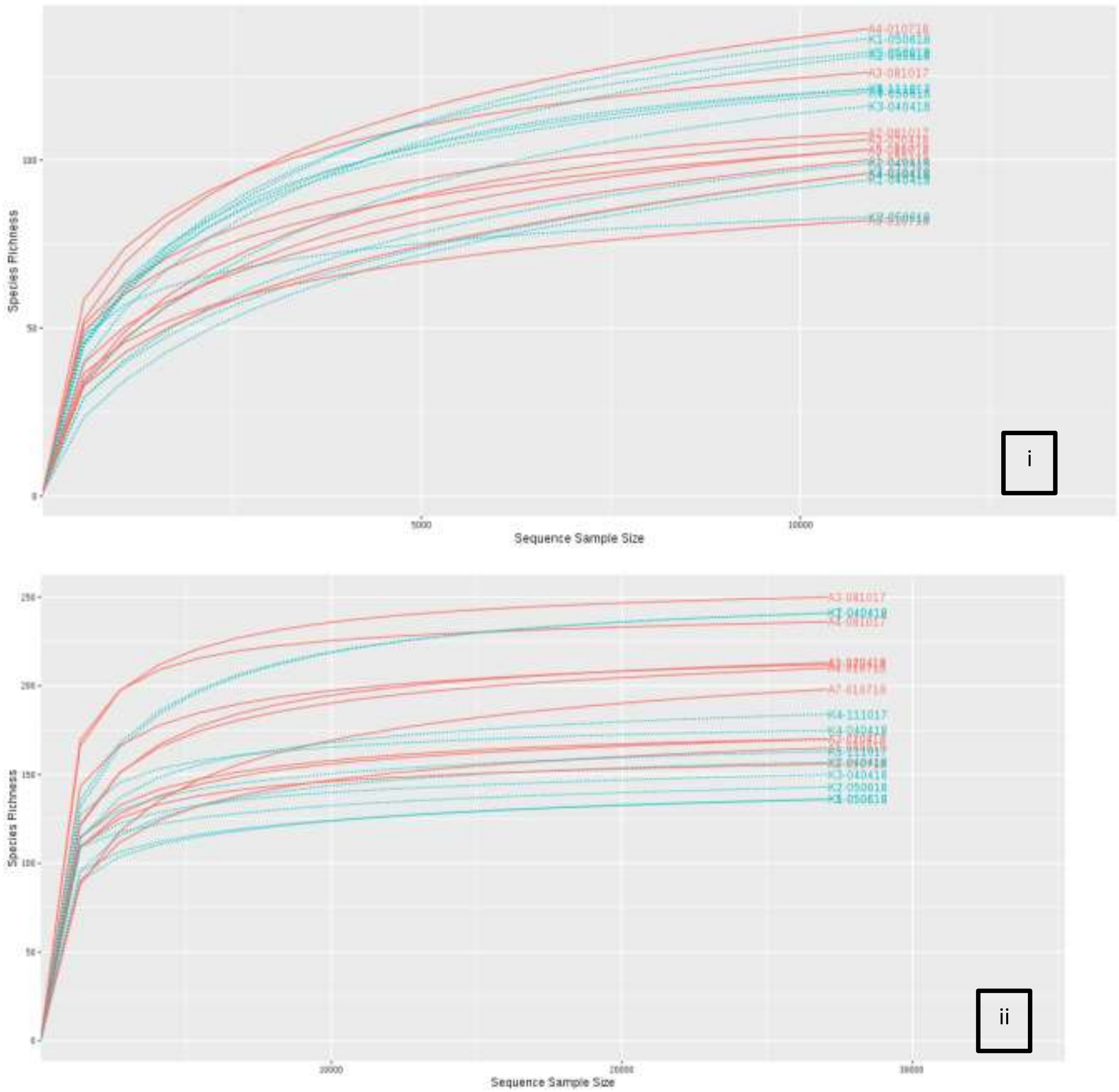

Figure S4b: Data Rarefaction Curves of (i) Bacterial and (ii) Fungal sequences
